# Recent advances in MOF-based single-atom photocatalysts for CO_2_ to solar fuel conversion under sunlight irradiation

**DOI:** 10.1039/d6sc00691d

**Published:** 2026-03-27

**Authors:** Adnan Majeed, Minh-Khoa Duong, Van-Duc Nguyen, Trong-On Do

**Affiliations:** a Department of Chemical Engineering, Laval University 1065 Avenue de la Médecine Quebec QC G1V 0A6 Canada Trong-On.Do@gch.ulaval.ca

## Abstract

Solar-driven photocatalytic CO_2_ conversion offers a sustainable route to address energy demand and carbon emissions. Metal–organic frameworks (MOFs) have emerged as promising photocatalyst platforms due to their tunable structures and well-defined coordination environments, while covalent organic frameworks (COFs) also offer ordered porosity and tunable electronic structures that support single-atom catalysts. Incorporation of single-atom active sites in MOFs and COFs further enhances site utilization, charge separation, and photocatalytic performance. This review offers a unified perspective on MOF-based single-atom photocatalysts for CO_2_ conversion by focusing on the synergistic interaction between atomically dispersed metal sites and MOFs. Unlike previous reports, it systematically compares different single-atom incorporation strategies and directly correlates atomic coordination environments with photocatalytic performance. By linking the structure, coordination, and activity, this work provides clear design guidelines for developing efficient and durable solar-driven CO_2_ reduction systems.

## Abstract 2

Rationally designed MOF-based single-atom catalysts enable efficient and selective solar-to-fuel CO_2_ conversion.

## Graphical abstract

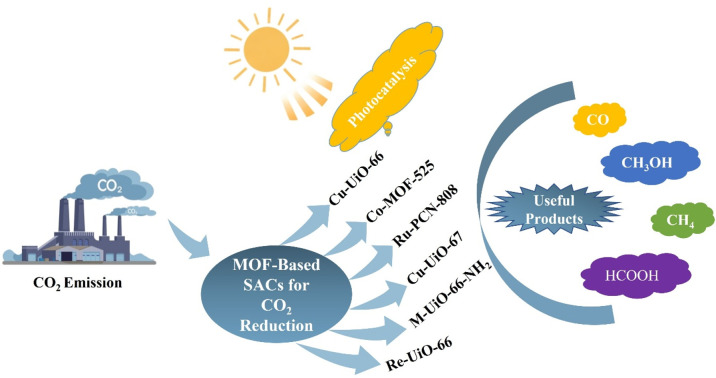

## 1 Introduction

Due to rapid growth and increasing industrialization, a serious energy crisis has emerged, leading to widespread water,^1,2^ soil,^3,4^ and air^5,6^ pollution.^7^ The energy demand is expected to escalate slightly; it will double by 2050 and triple before the end of this century.^8,9^ Such growth will place enormous strain on existing energy systems. Although fossil fuels contribute heavily to meeting the fuel requirements of the world, their continuous use contributes largely to rising global warming.^10^ Moreover, owing to the ample use of mineral fuel, greenhouse gases such as carbon dioxide (CO_2_) have escalated significantly.^11^ The International Energy Agency believed that CO_2_ emissions from energy production would peak in about 2025 based on the recent publication of World Energy Outlook 2023.^12,13^ Despite the projected peak, atmospheric CO_2_ concentrations continued to increase, surpassing 430 ppm by the end of April 2025, with an estimated growth rate of more than 2 ppm per year.^4,14,15^ This rapid rise in CO_2_ plays a significant role in enhancing the process of global warming and other natural disasters like the melting of polar ice, acidification of the oceans, and climate instability.^16,17^ Consequently, the urgent reduction of atmospheric CO_2_ concentration has become a critical global priority.^18^ To address this dilemma, much effort has been invested to establish efficient CO_2_ transformation and reduction methods, *i.e.*, thermocatalysis,^19^ electrocatalysis,^20^ and photocatalysis.^22^

**Fig. 1:**
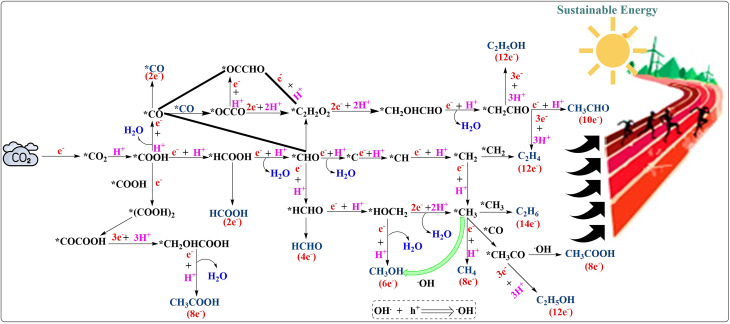
Schematic representation of the possible photocatalytic CO_2_ reduction pathways producing C_1_ and C_2_^+^ products. Redrawn using ChemDraw.^28,29,35^

The reduction of CO_2_ itself is quite difficult because of the large amount of energy needed to overcome the extremely stable C=O bond with a bond energy of around 805 kJ mol^−1^.^21^ There are many methods for the reduction of CO_2_, but using a photocatalyst is quite appealing due to an eco-friendly process, in which CO_2_ can be reduced to various value-added products^22^ such as HCOOH,^23^ CO,^24^ CH_4_,^25^ CH_3_OH,^26^*etc.* The use of sunlight is especially promising, as it is abundant and readily available.^27^ In nature, photosynthesis is a well-known biological process that reduces CO_2_ under sunlight, converting it into chemical forms. In photocatalytic reduction reactions, the reduced product can be C_1_ or C_2_^+^ molecules, as presented in Fig. 1.^28^ Each molecule faces its own thermodynamic and kinetic barriers during reduction.^29^ The process is quite efficient depending upon the nature of the photocatalyst used, which may increase the yields.^30^ The most important factors are the efficiency of absorption of photons and the transfer characteristics of charges in the photocatalyst.^31^ Despite major efforts to optimize these factors, the overall activity of photocatalytic materials remains insufficient to meet the growing demands of the environmental and energy sectors.^32^ Consequently, for the evolution of novel photocatalytic materials, it would be imperative to combine high absorption and efficient charge separation.^33,34^

Single-atom catalysts (SACs) have recently attracted much interest as highly promising photocatalysts owing to their distinctive atomic properties, such as maximum metal utilization efficiency and high catalytic activity.^36,37^ Single atoms (SAs) in the photocatalytic system can also create isolated reaction sites, which can not only provide more active sites but also increase light harvesting and charge separation.^38^ These findings collectively confirm that isolated metal sites enhance both light absorption and charge transfer, which are crucial for high-performance SA photocatalysis. In addition, their coordinatively unsaturated configuration provides well-defined active sites for reactant adsorption and activation. Such features enable improved photocatalytic performance while retaining high atomic utilization efficiency.^39^ SAC models were first developed by Zhang *et al.* to describe the high activity and stability of isolated Pt atoms on iron oxides, attributable to metal-support interactions.^40,41^ The unsaturated sites, quantum size effects, and strong metal-support interactions of atomic-sized SACs enable catalytic activity similar to that of homogeneous catalysts and heterogeneous stability.^42,43^ However, it should be pointed out that atomic-scale metal dispersal could raise surface free energy owing to the inherent metallic atom clustering nature.^44,45^ Hence, the process of immobilizing individual atoms on practical surfaces is now a current research field of interest in chemistry, environmental science, and materials science.^46^ Therefore, supporting SAs on carefully designed substrates is crucial to fully exploit the catalytic capacity of SACs.^47,48^

**Fig. 2:**
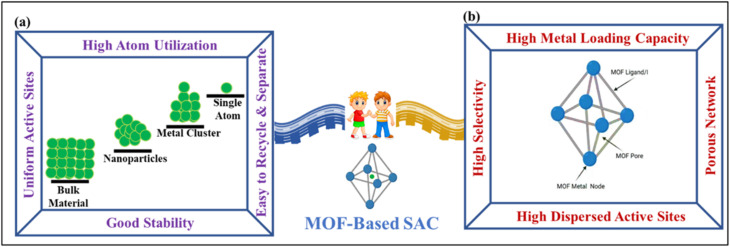
Schematic diagram of (a) single-atom catalysts, and (b) MOF properties.

In recent years, metal–organic frameworks (MOFs) have emerged as highly effective supports for SACs because of their structural flexibility and synergistic catalytic properties.^49^ The high density of coordination sites within MOF architectures allows SACs to be anchored at fixed positions, effectively preventing atom migration and aggregation.^50,51^ Supporting SACs on MOFs not only reduces aggregation but also promotes synergistic catalysis in the composite material.^52^ Consequently, SACs derived from MOFs provide an efficient bridge between homogeneous and heterogeneous catalysis,^53^ as illustrated in Fig. 2. In addition to MOFs, covalent organic frameworks (COFs) have recently attracted attention as alternative platforms for single-atom photocatalysts due to their well-defined porous structures, crystallinity, and tunable π-conjugated backbones. Their predesignable architectures enable precise control over light absorption, electronic structure, and charge separation behavior, making them suitable for stabilizing isolated metal sites for photocatalytic CO_2_ reduction.^54^ The incorporation of single metal atoms into COFs not only facilitates CO_2_ activation but also promotes efficient electron transfer from the conjugated network to the catalytic centers. Moreover, the photocatalytic performance of COFs can be systematically tuned through the selection of precursors, linkage chemistry, framework topology, and the nature of the anchored metal atoms.^55^ However, despite these advances, MOF-based systems remain the primary focus of this review due to their diverse coordination environments and well-established structural tunability. Nonetheless, recent reviews have highlighted some progress in MOF-based photocatalysts for CO_2_ reduction. However, limited attention has been given to MOF-based SACs (MOF@SACs), including both MOF-supported and MOF-derived structures, in the context of photochemical energy conversion. Building on our previous review,^18^ this work strives to illustrate structure–activity relationships and outline future directions for MOF-based single-atom catalysts toward efficient and sustainable solar-driven applications and product conversion. This review is solely focused on the reduction of CO_2_ by MOF@SACs.

## 2 Fundamentals of MOF-based single-atom photocatalysts for CO_2_ reduction

**Fig. 3:**
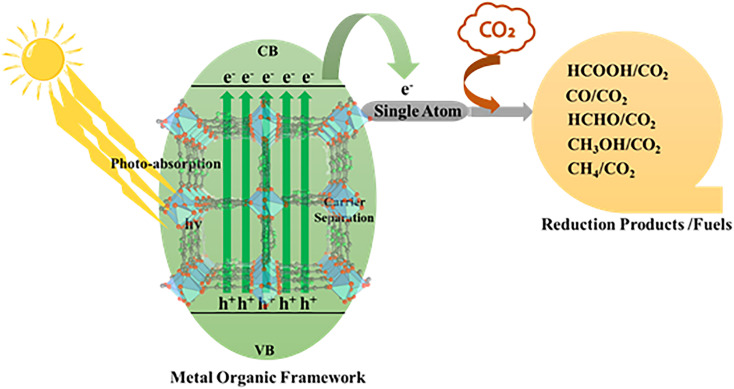
Schematic of MOF-based single-atom photocatalytic CO_2_ reduction under solar light irradiation.

In MOF-based single-atom photocatalysts, this process is governed by the synergistic interplay between the light-harvesting framework and atomically dispersed metal active sites (Fig. 3),^57^ which facilitate efficient charge transfer and suppress recombination.^56^ The MOF-based photocatalytic CO_2_ reduction reaction is catalyzed by a series of elementary reactions, similar to those in heterogeneous catalysis that involve the adsorption of CO_2_, surface reactions by charge transfer, and desorption of products.^57,58^ CO_2_ adsorption is a step that cannot be underestimated, because the linear geometry of the molecule and the high thermodynamic stability of CO_2_, which is due to strong C=O double bonds, make the activation of CO_2_ intrinsically hard.^59,60^ The well-defined pores, high surface area, and adjustable chemical environment in MOF-based photocatalysts help enrich CO_2_ around active sites to reduce diffusion barriers and maximize local reactant concentration.^61,62^ A CO_2_ activation reaction has to ensure that the conduction band edge of the photocatalyst is more negative than the CO_2_ reduction potential, whereas the valence band edge is sufficiently positive to maintain water oxidation.^63,64^ Proper band alignment is therefore essential to ensure that photogenerated carriers possess adequate driving force to initiate and sustain redox reactions.^65^ The activation of CO_2_ at the reaction interface typically occurs *via* interaction with *CO_2_ (commonly referred to as the rate-limiting step), which has a very negative reduction potential.^66^ The molarity of many photocatalysts is insufficient to activate this one-electron step effectively; therefore, proton-assisted multi-electron transfer mechanisms are preferred, which decrease the energy barrier for the activation of CO_2_.^67^ Subsequent reaction pathways diverge toward the formation of C_1_ or C_2_^+^ products (Fig. 1). C_1_ products typically originate from key intermediates such as *COOH and *OCHO, whereas C_2_^+^ products require C–C coupling steps involving species such as *CO, *CHO, or *COCO.^68,69^ Even though C_2_^+^ products are more energy-rich and possess more practical utility, their production is kinetically more difficult and necessitates the fine control of intermediate adsorption strength, surface mobility, and coupling probability.^64,70^

The single-atom (SA) active sites attached to MOFs are important in controlling such elementary steps.^46,71^ They have the benefit of allowing the control of the local electronic structure and coordination environment with a high level of precision, which directly affects the CO_2_ adsorption strength, the stability of intermediates, and charge-transfer kinetics.^72^ Besides, the charge transfer of SAs with the MOF host using metal–ligands can create new electronic states to absorb more visible light and improve the separation of charge carriers generated by photoexcitation.^73^ Notably, the atomic dispersion of the metal center optimizes the utilization of the metal and avoids the formation of metallic clusters, which are likely to be recombination centers.^74,75^ These properties enable MOF-based single-atom (MOF@SA) photocatalysts to act as highly efficient platforms towards selective CO_2_ activation and conversion.^76^

The porous structure of MOFs offers spatial confinement effects that can stabilize chemical intermediates and encourage particular reaction routes in addition to electrical regulation. Fine control over reaction kinetics and product selectivity is made possible by the diffusion of reactants and intermediates within MOF channels and customized coordination environments surrounding SAs.^77,78^ However, excessive intermediate binding can delay product desorption and block sites, emphasizing the significance of balancing adsorption and desorption.^79^ Thus, SA photocatalysts based on MOFs have to be rationally designed, that needs to take into account light absorption, charge separation, band alignment, active-site accessibility, and intermediate binding strength.^80^ Through combining tunable MOF structured frameworks with atomically dispersed metal sites, the systems offer an unprecedented platform to resolve the inherent drawbacks of CO_2_ activation, rejection of charge recombination, and reaction directionalities to products of interest.^81^ The knowledge of these core concepts forms a good basis for rational development of next-generation MOF@SA photocatalysts, which are effective in reducing CO_2_ under sunlight. SA photocatalysts based on MOFs have clear benefits over traditional ones due to the ability to control the binding energy of the reaction intermediate by the number of metal atoms in the crystal, which increases the catalytic efficiency. Nonetheless, overly powerful binding of intermediates is counterproductive because it prevents the desorption of products and reduces the turnover. The development of an optimal, moderate binding strength in MOF@SACs architectures, thus, is a major challenge.

## 3 Synthetic strategies for single-atom incorporation in MOFs

Both homogeneous and heterogeneous catalysts can be used in both industrial practice and fundamental research. In homogeneous catalysis, the catalyst and the reactants are in the same phase, which makes the active site well-defined and the reaction mechanism clear; however, poor stability and separation make such an application not practicable.^82,83^ In contrast, heterogeneous catalysts are more stable and easily recoverable; but in many cases, their catalytic activity is complicated by surface heterogeneity. This heterogeneity is due to different atomic structures, defects, and variation of particle sizes.^84^ It is so complex that it is difficult to spot the same active sites, and comparison of catalytic performance is then not reliable. In order to overcome these shortcomings, converting metal nanoparticles into single-atom catalysts (SACs) has been a viable approach.^85^ With the combination of molecular-level specificity of homogeneous catalysts and the stability and reusability of heterogeneous systems, SACs allow customizable reactivity, enhanced metal productivity, and atomic-scale photocatalysis.^86^ Overall, recent years have seen massive progress in the field of synthetic chemistry, which has greatly increased the pace at which SA catalysts can be developed.^43,71,87,88^ These active sites have a high inherent surface energy and therefore can migrate and cluster or form nanoparticles during synthesis and further processing due to a large quantity of active sites per mole compared to bulk materials.^89^

Supported catalysts often have a blend of metal nanoparticles and atomically dispersed metal atoms, and high and uniform loadings of active sites are hard to reach.^90^ Scientists have thus concentrated on synthetic techniques, which maintain the distant togetherness of metal precursors, avoid agglomeration of these during thermal or chemical treatments, and provide sufficient anchoring spots to retain solitary metal atoms.^91^ The introduction of MOFs as host matrices with added guest species is an effective way of increasing the charge-carrier separation in photocatalysis. This is enhanced by the interfacial interaction or heterojunctions, which develop internal electric fields that facilitate effective separation and transport of charges. MOF-derived carbon supports are promising when constructed on mixtures of MOFs and porous crystalline materials.^92^ They are self-assembled using metal ions/clusters with organic linkers, thereby offering high surface area, defined pores, tunable chemistry, and numerous coordination sites.^93^ MOF-based carbon materials have been demonstrated to be superior SAC supports, which enhance catalyst stability, ease recovery, and allow SACs with homogeneous and heterogeneous catalysis to be combined.^41,94^

Four major methods are used to produce MOF@SA catalysts: intrinsic linker stabilization, post-synthesis ligand modification, metal node stabilization, and nanopore confinement.^95^ SAs may be immobilized through coordination with metal nodes or organic ligands, whereas confinement within MOF pores further suppresses aggregation by physically separating metal precursors of appropriate size. The combination of these techniques enables this to be controlled in terms of atom dispersion, stability, and catalysis.^96^ Heterogeneous catalysis is said to involve coordinatively unsaturated metal sites in MOFs, which are considered to be the chief active centers. Their electrophilic nature imparts Lewis acidity, enabling efficient electron acceptance and favorable interactions with nucleophilic or reactive species, thereby enhancing catalytic performance.^97^ Other sites that can be post-synthetically modified to add more catalytic centers are provided by unsaturated metal ions or cluster nodes.^98^ This is particularly useful with MOF-based photocatalysis, in which it enhances light absorption, charge separation, and surface redox reactions. The use of these unsaturated metal nodes provides an opportunity to allow isolated metal atoms to increase the catalytic ability of MOFs.^99^ Node-anchored species can remain stably dispersed at the atomic level without aggregating into nanoparticles, due to strong coordination bonds between the SAs and the inorganic metal clusters.^100^ This anchoring strategy also optimizes the local environment of the active sites, thereby facilitating the photocatalytic activation of CO_2_.^81,101^ Several strategies have been developed to modulate the local structure of SA active sites and evaluate their catalytic performance for CO_2_ conversion. These approaches include the design of molecular catalysts, carbonization of functionalized precursors, and tuning the coordination number of metal centers supported on MOFs.^102^ The coordination environment of SAs plays a crucial role in determining the catalytic activity of a material.^103^ When SAs are anchored between the layers of a catalyst, they can promote charge separation and enable efficient interlayer charge transfer, which improves overall catalytic performance.^104^

### 3.1 Metal node anchoring strategy

**Fig. 4:**
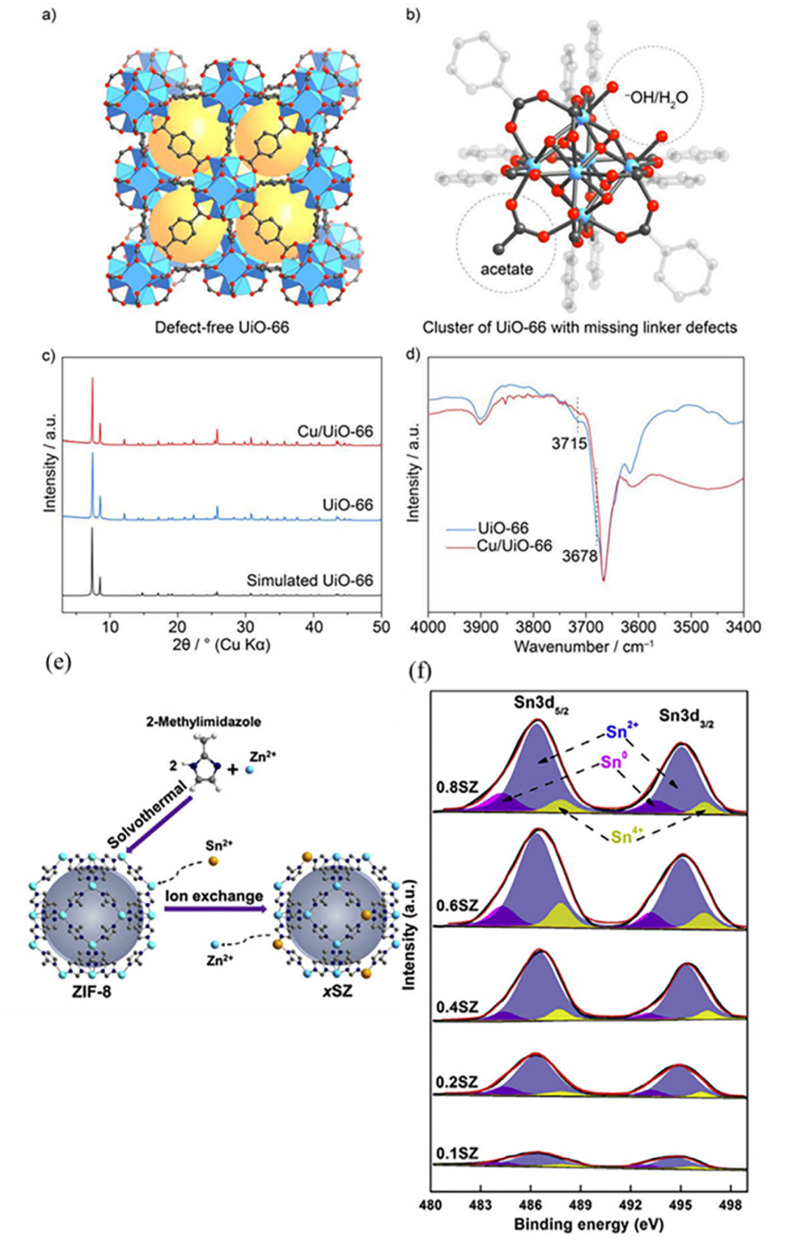
(a) Crystal structure of defect-free UiO-66 (yellow spheres indicate pore space). (b) Zr_6_-oxide cluster showing defect sites capped by –OH/–OH_2_ and acetate groups (C, black; O, red; Zr, blue; H are omitted). (c) PXRD patterns of UiO-66 and Cu/UiO-66 compared with the simulated UiO-66 pattern, confirming framework integrity. (d) DRIFTS spectra of UiO-66 and Cu/UiO-66 showing changes in –OH vibrations after Cu anchoring. Reproduced with permission from ref. 108. (Copyright 2019 American Chemical Society). (e) Schematic preparation of Sn-doped ZIF-8. (f) Sn 3d XPS spectra of Sn-ZIF-8 with different tin contents. Reproduced with permission from ref. 109. (Copyright 2020 Wiley-VCH).

Metal node anchoring represents an effective strategy for stabilizing single atoms in MOFs, as summarized in recent studies on MOF-based single-atom catalysts.^105–107^ Metal clusters at MOF nodes often contain coordinatively unsaturated sites terminated by inorganic ligands such as –OH or –OH_2_. These oxygen-containing groups provide strong coordination environments for anchoring isolated metal atoms in the node region rather than on organic linkers. Abdel-Mageed and co-workers demonstrated that Cu single atoms can be anchored at defective Zr_6_-oxo nodes of UiO-66 through coordination with terminal –OH or –OH_2_ ligands. Spectroscopic and microscopic characterization confirmed that the Cu species remain atomically dispersed without aggregation, resulting in fully exposed and stable active sites^108^ (Fig. 4). Geng and collaborators utilized a node metal ion-exchange strategy that has been demonstrated in ZIF-8, where partial replacement of Zn^2+^ ions by Sn^2+^ ions leads to the formation of isolated Sn species at the metal nodes, while retaining the structural integrity of the framework.^109^

**Fig. 5:**
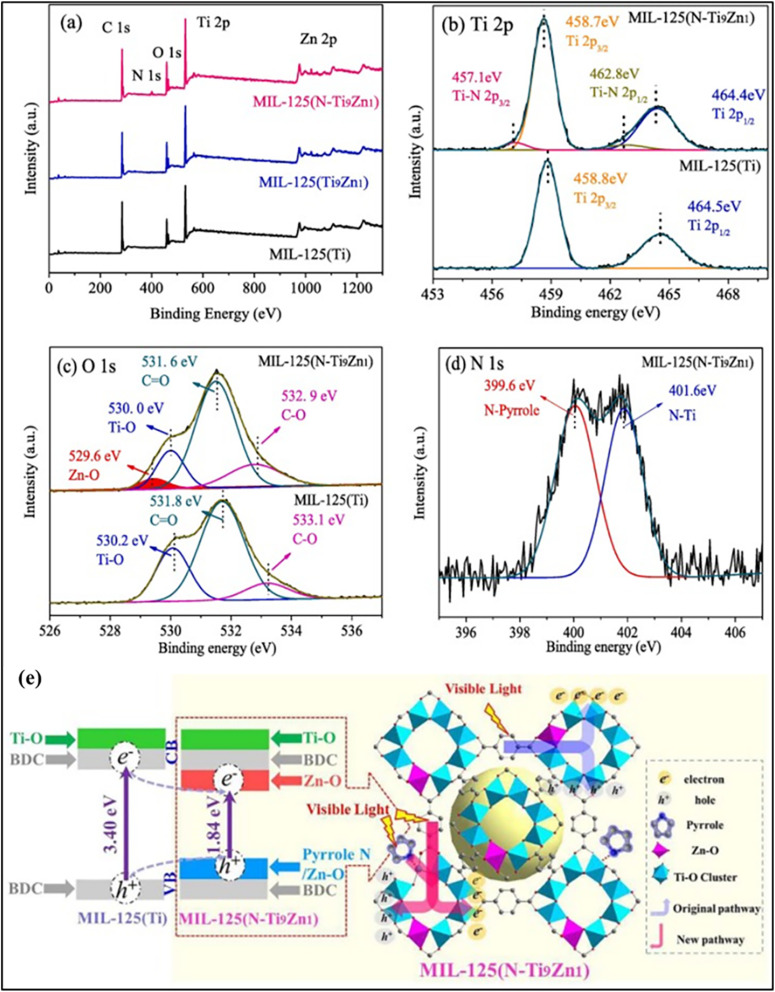
(a) XPS survey spectra of MIL-125(Ti), MIL-125(Ti_9_Zn_1_), and MIL-125(N–Ti_9_Zn_1_). High-resolution (b) Ti 2p and (c) O 1s XPS spectra of MIL-125(Ti) and MIL-125(N–Ti_9_Zn_1_) and (d) N 1s XPS spectra of MIL-125(N–Ti_9_Zn_1_). (e) Schematic illustration of photogenerated charge separation and band-structure modulation induced by Zn and N doping at the MOF metal nodes. Reproduced with permission from ref. 110. (Copyright 2020 Elsevier).

Zhu Gao and co-workers developed an *in situ* co-doping strategy for MIL-125(Ti) by introducing Zn(ii) and pyrrolic N during hydrothermal synthesis. Zn partially substituted Ti at the metal nodes, generating non-equivalent active sites, while pyrrolic N coordinated with Ti clusters to form Ti–N bonds. XPS confirmed Ti^3+^ formation, oxygen vacancy generation, and N–Ti coordination (Fig. 5a–d). A slight reduction in surface area indicated node-related defects. The node-anchored Zn and N species improved charge separation and transfer, as evidenced by reduced PL intensity, lower charge-transfer resistance, increased photocurrent, and stronger ESR signals. As a result, MIL-125(N–Ti_9_Zn_1_) exhibited a photocatalytic CH_3_CHO degradation rate nearly ten times higher than that of pristine MIL-125(Ti), demonstrating the effectiveness of metal-node anchoring in enhancing photocatalytic performance. Fig. 5e shows the schematic illustration of photogenerated charge separation and band-structure modulation induced by Zn and N doping at the MOF metal nodes.^110^

**Fig. 6:**
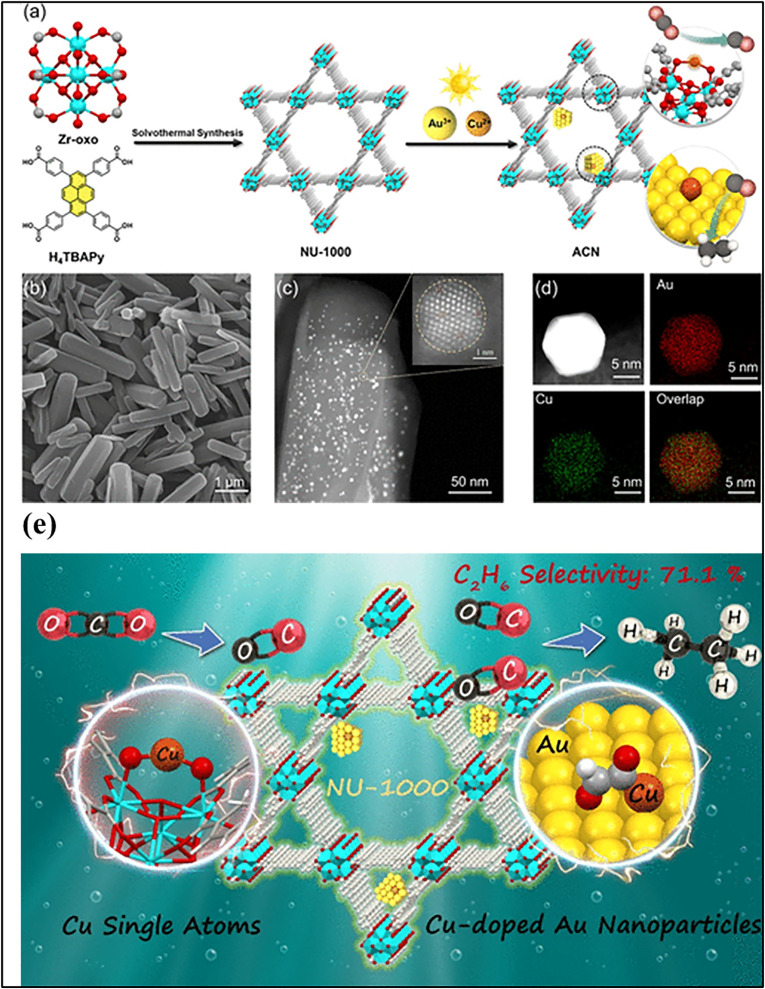
(a) Schematic of the synthesis of (AuCu-NU-1000) ACN, showing CO_2_ conversion sites. (b) SEM image of ACN. (c) HAADF-STEM image of ACN with a zoomed-in view of a single nanoparticle. (d) HAADF-STEM of an enlarged nanoparticle with elemental maps of Au, Cu, and their overlap. (e) Schematic of the overall catalytic mechanism of a single-atom-based MOF. Reproduced with permission from ref. 111. (Copyright 2025 American Chemical Society).

**Fig. 7:**
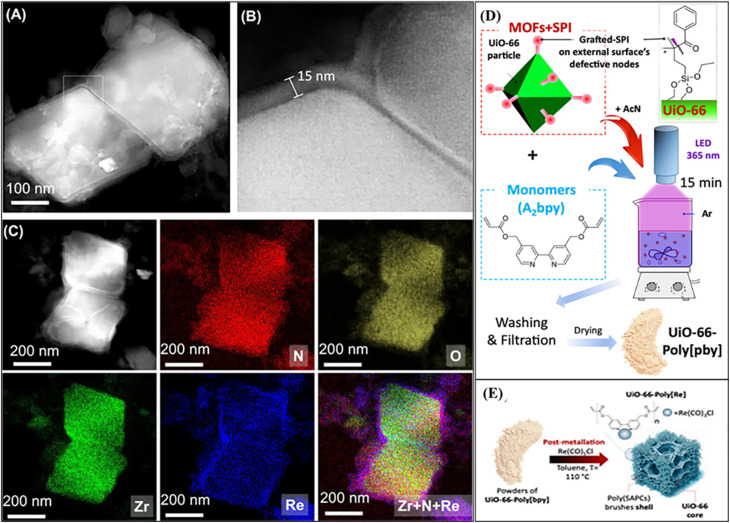
(A) HAADF-STEM of UiO-66-poly[Re] showing a 15 nm poly[Re] shell. (B) Zoomed view of the shell. (C) EDX elemental maps for N (red), O (yellow), Zr (green), and Re (blue). (D) Photopolymerization of A_2_bpy on UiO-66-SPI. (E) Functionalization of UiO-66-poly[bpy] with Re(CO)_5_Cl to form UiO-66-poly[Re]. Reproduced with permission from ref. 112. (Copyright 2025 American Chemical Society).

In another study, the Di Chen group introduced photo-deposition as an effective strategy for incorporating single-atom sites into MOFs, particularly Zr-based MOFs such as UiO-66 NU-1000, and MOF-808, which possess coordinatively unsaturated Zr_6_-oxo nodes terminated by –OH/–OH_2_ ligands. Under light irradiation, metal precursors (*e.g.*, Cu^2+^, Fe^3+^, Ni^2+^, and Au^3+^) can be reduced and stabilized as atomically dispersed species at the metal nodes, typically with loadings below 1 wt%, as confirmed by HAADF-STEM (Fig. 6). The high surface area (often >2000 m^2^ g^−1^) and mesoporous channels (∼2–3 nm) of these MOFs further suppress aggregation and enable the confined integration of single atoms and atomically modified metal nanoparticles in proximity. This node-anchored photodeposition approach provides precise control over atomic dispersion and multisite construction, offering a versatile synthetic pathway for MOF-based single-atom photocatalysts.^111^ Youssef *et al.* reported a method for incorporating SACs into MOFs *via* surface functionalization and postmetalation. UiO-66 was first modified with a silane-based photoinitiator, followed by surface-initiated photopolymerization of bipyridine derivatives to form a polymer shell. Postmetalation with Re(CO)_5_Cl generated well-dispersed rhenium single-atom sites anchored on the MOF surface. The HAADF-STEM and EDX analyses confirmed the formation and uniform distribution of the SA shell around the UiO-66 particles (Fig. 7A–C). This hybrid material retained the MOF's structural integrity and porosity (Fig. 7D and E), showed enhanced CO_2_ adsorption and charge separation, and exhibited high photocatalytic activity, highlighting the potential of surface-initiated polymerization and postmetalation for preparing efficient single-atom MOF catalysts.^112^

### 3.2 MOF-derived carbon-based single-atom catalysts

The preparation of MOF-derived carbon supports involves the synthesis of suitable MOF precursors followed by controlled pyrolysis under a defined atmosphere. During thermal treatment, organic ligands decompose to form a porous carbon matrix, while coordinated metal centers evolve into atomically dispersed M–N_*x*_ active sites.^113^ The pyrolysis temperature plays a critical role: moderate temperatures enhance graphitization and conductivity, whereas excessively high temperatures may reduce heteroatom content (*e.g.*, nitrogen) and promote metal aggregation. Therefore, optimization of carbonization temperature is essential to balance conductivity and active site preservation. For Zn-containing MOFs, temperatures above 900 °C are typically used to remove Zn species and generate metal-free porous carbon. In contrast, transition-metal-based systems are commonly carbonized at 700–1000 °C to stabilize isolated metal atoms (*e.g.*, Fe–N_4_, Ni–N_4_, and Pt–N_4_) within nitrogen-doped carbon frameworks. The carbonization atmosphere also influences the final structure: inert gases (Ar or N_2_) prevent oxidation, while NH_3_ can enhance nitrogen doping and increase coordination sites.^114,115^ Using MOF-derived carbon-based SACs has emerged as a dominant strategy for constructing highly stable and conductive SACs. They effectively overcome the structural instability of pristine MOFs at high temperatures and in harsh chemical environments.^53,116^ As a result, they exhibit enhanced stability and promising catalytic performance. Their morphology and pore structure can be precisely controlled through MOF precursor design, and the coordination environment of metal sites can be tuned *via* linkers and pyrolysis conditions. The inherent porous architecture provides high surface area and efficient mass and charge transport, while MOF cavities and unsaturated sites enable construction of well-defined single-atom active centers.^113^ Additionally, *in situ* characterization is needed to accurately probe active sites under working conditions and guide the design of more efficient MOF-derived SACs.^117^

**Fig. 8:**
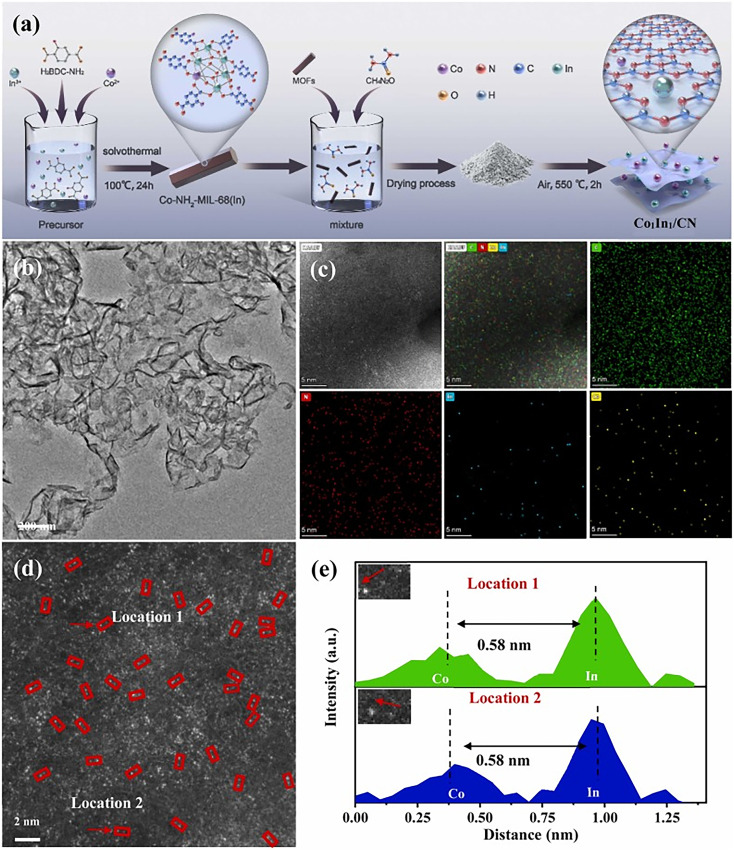
(a) Schematic of Co_1_In_1_/CN synthesis. (b) TEM, (c) HAADF-STEM with elemental mapping, (d) AC-STEM, and (e) corresponding atomic intensity profiles. Reproduced with permission from ref. 118. (Copyright 2025 Elsevier).

Biao Hu *et al.* demonstrated a MOF-derived strategy for incorporating single and dual metal atoms into a photocatalytic carbon nitride matrix. Using NH_2_-MIL-68(In) as a precursor, atomically dispersed Co and In sites were successfully anchored on a CN support *via* a straightforward pyrolysis approach. High-resolution HAADF-STEM, EXAFS, and XANES analyses confirmed the formation of Co–N_4_ and In–N_5_ coordination structures without nanoparticle aggregation (Fig. 8). The Co_1_In_1_ system derived from MOF precursors exhibits enhanced photocatalytic performance, where the MOF structure enables uniform dispersion of dual-metal sites and facilitates efficient charge transfer.^118^

### 3.3 Ultrathin MOF nanosheet engineering

**Fig. 9:**
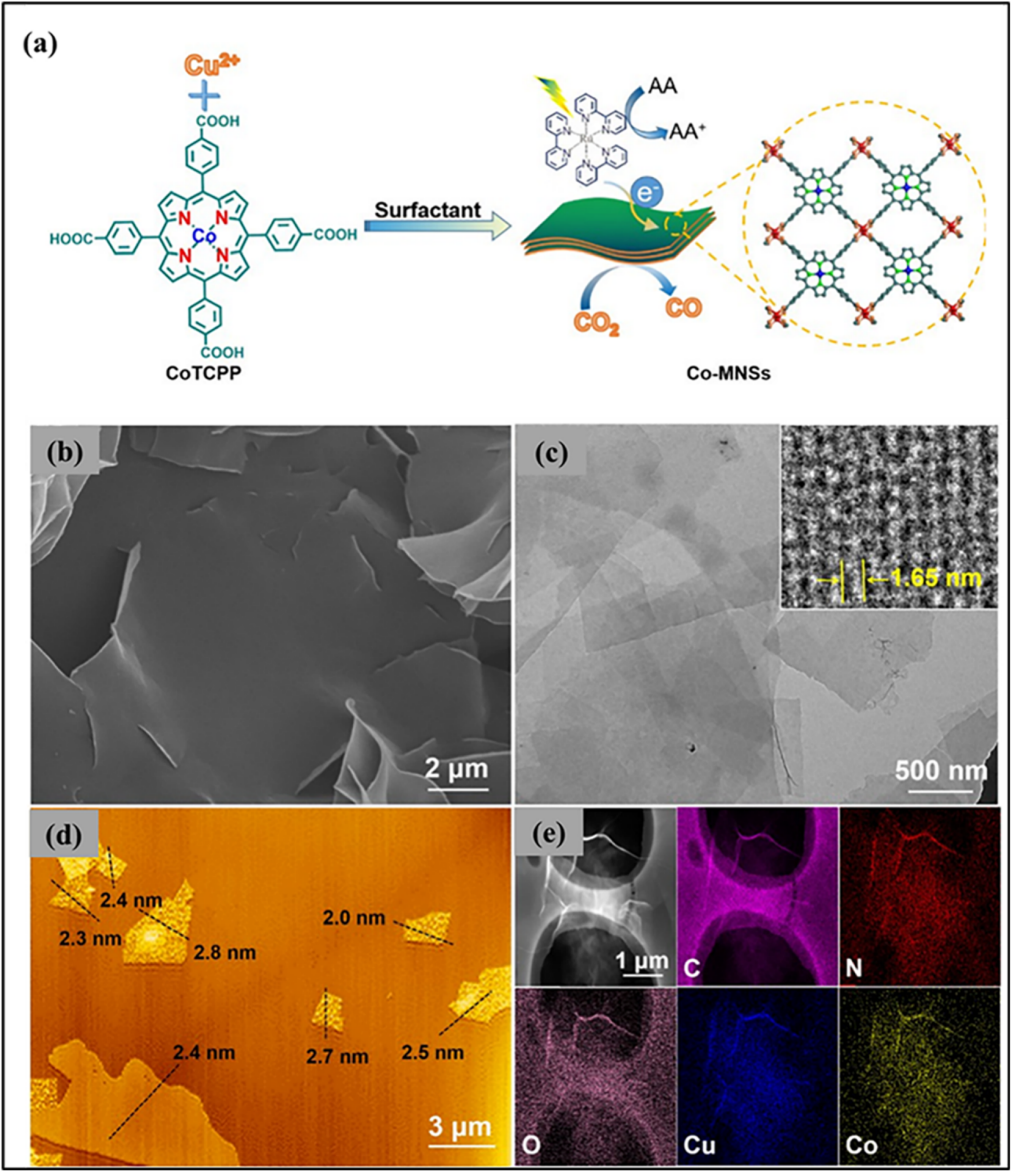
(a) Schematic of ultrathin Co single-atom MOF nanosheet synthesis. (b and c) SEM and TEM images. (d) AFM height profile. (e) HAADF-STEM image with EDS elemental mapping. Reproduced with permission from ref. 120. (Copyright 2023 Springer Nature).

The ultrathin nanosheets of MOFs have open pores on both sides, high accessibility of active sites, and shorter pathways for charge diffusion.^119^ Quan Zuo and colleagues reported an effective strategy for stabilizing single atoms using ultrathin MOF nanosheets as supports.^120^ Compared with bulk MOFs, two-dimensional nanosheets provide open pores on both sides,^121^ enabling high SA loading and improved contact with CO_2_ molecules, while the ultrathin thickness shortens charge diffusion pathways and suppresses electron–hole recombination.^122^ Through a bottom-up synthetic approach, Co single atoms were pre-coordinated within porphyrin linkers and assembled into ultrathin MOF nanosheets (Co-MNSs), avoiding the low yield and complexity of exfoliation methods (Fig. 9a). The resulting Co-MNSs exhibited an ultrathin thickness of ∼2.4 nm with micron-sized lateral dimensions (Fig. 9b–d), delivering abundant exposed active sites. As a result, Co-MNSs achieved efficient visible-light-driven CO_2_-to-CO conversion with high activity and selectivity, demonstrating the advantages of ultrathin MOF nanosheets for single-atom photocatalysis.^120^

### 3.4 Pre-synthetic incorporation strategies

**Fig. 10:**
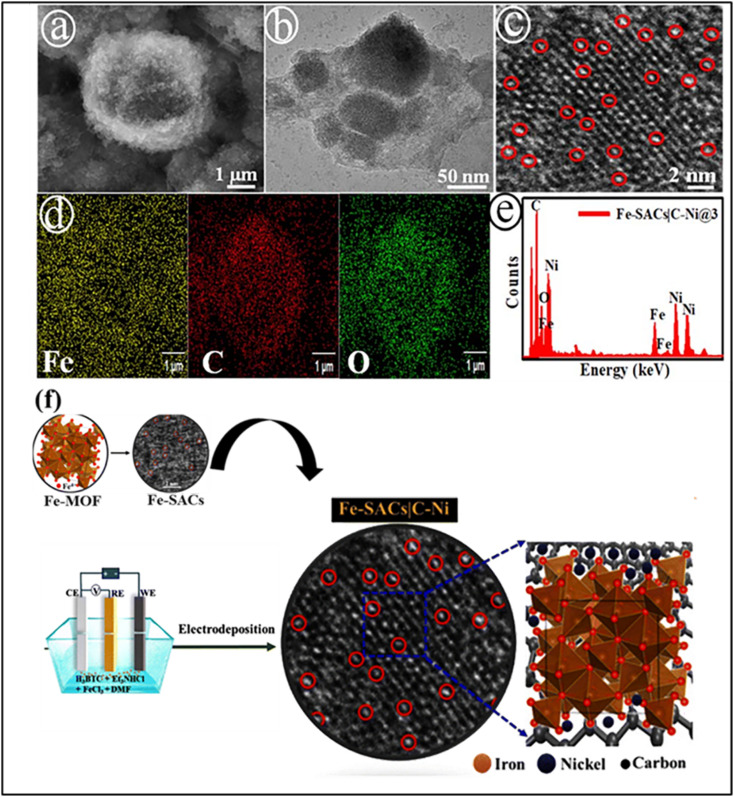
SEM (a), TEM (b), and high-resolution TEM (c) images of the catalyst; the inset highlights the dispersed Fe single atoms on the C–Ni nanosheets. Elemental mapping (d) and the corresponding EDX spectrum (e) confirm the elemental distribution of the Fe-SACs/C–Ni@3 electrode along with the Fe 2p spectrum and (f) a schematic illustration of the pre-synthetic electrodeposition strategy used for MOF-based single-atom catalysts. Reproduced with permission from ref. 123. (Copyright 2024 Royal Society of Chemistry).

Pre-synthetic incorporation of single-atom catalysts into MOFs allows uniform atomic dispersion and strong coordination with organic linkers or metal nodes, effectively suppressing aggregation and improving catalytic performance for CO_2_ reduction. Unlike post-synthetic methods, the Shankar group introduced metal precursors during MOF formation, enabling better control over the coordination environment and electronic structure. In that study, electrodeposition-assisted MOF growth was achieved, in which Fe single atoms are incorporated onto carbon-modified nickel nanosheets (C–Ni NSs) through an electrochemical process. In this method, Fe^3+^ ions are reduced to Fe^2+^ in the presence of benzenetricarboxylate (BTC^3−^), promoting MOF growth directly on the electrode surface, as confirmed by various characterization techniques (Fig. 10a–f). The *in situ* coordination stabilizes isolated metal sites and strengthens metal-support interactions, resulting in durable and efficient catalytic centers, suggesting the potential of this approach for designing MOF-based SACs for CO_2_ reduction.^123^

### 3.5 Linker engineering and metallation

**Fig. 11:**
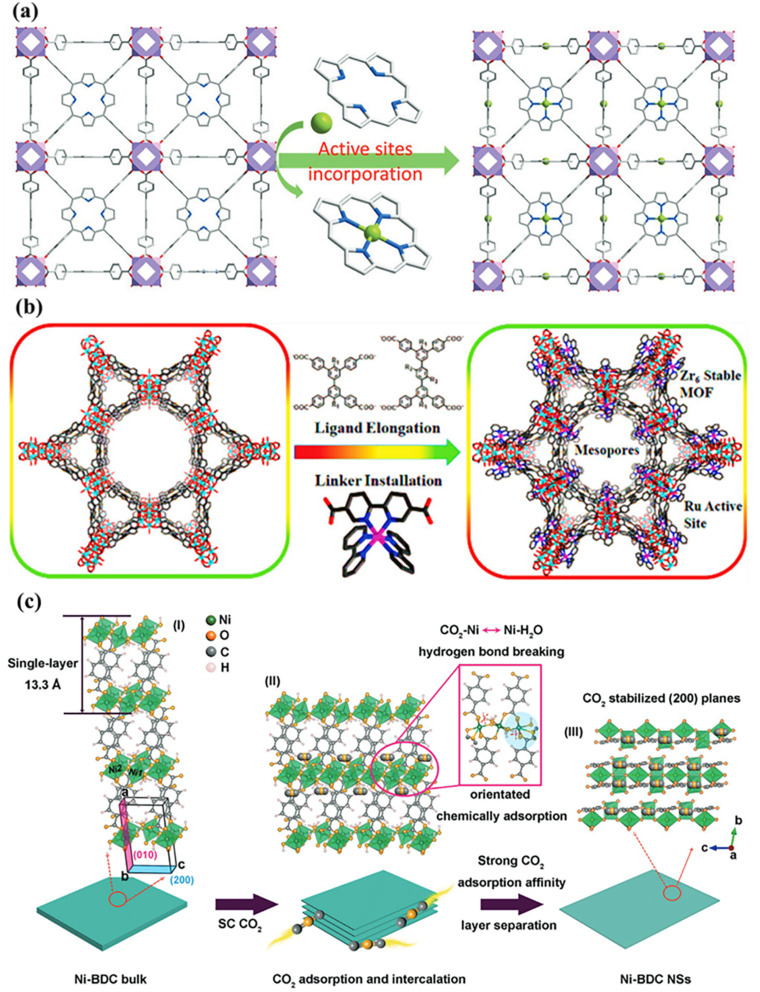
(a) 3D network of MOF-525-Co, showing its highly porous framework with incorporated single Co active sites. Reproduced with permission from ref. 51. (Copyright 2016 Wiley-VCH). (b) Schematic illustration of PCN-808-BDBR synthesis, where [Ru(bpy)_3_]^2+^ active sites are installed on the organic linkers, forming a stable Zr_6_-based MOF with mesopores. Reproduced with permission from ref. 126. (Copyright 2020 American Chemical Society). (c) Schematic illustration of the formation of Ni-BDC nanosheets (NSs). (I) Three-dimensional crystalline structure of Ni-BDC viewed along the [010] direction, illustrating the layer stacking arrangement. Nickel, oxygen, carbon, and hydrogen atoms are represented in green, orange, gray, and pink, respectively. (II) CO_2_ molecules replace coordinated H_2_O and bind to Ni^1^ sites, breaking the interlayer hydrogen bonding. (III) The chemically adsorbed CO_2_ stabilizes the exfoliated single-layer Ni-BDC nanosheets, exposing the active (200) planes. Reproduced with permission from ref. 127. (Copyright 2021 WILEY-VCH).

In addition to metal clusters, the organic linkers in MOFs possess strong anchoring ability and can effectively coordinate with single-atom metal sites.^100^ Porphyrins, which contain four pyrrolic nitrogen atoms arranged in square-planar or tetrahedral geometries, are especially effective for trapping metal SAs through coordination interactions arising from their lone-pair electrons.^124^ Zhang *et al.* reported that exciton migration in natural photosynthesis mainly occurs in highly ordered porphyrin-like pigments. Inspired by this, MOF-525, with the formula Zr_6_O_4_(OH)_4_(TCPP-H_2_)_3_ was synthesized. This MOF integrates Zr_6_ clusters and porphyrin units into a 3D network. Coordinatively unsaturated Co sites were introduced into the porphyrin units to form MOF-525-Co (Fig. 11a). In this structure, each Co active site is simultaneously exposed to CO_2_ molecules while being spatially isolated, preventing aggregation. These unsaturated Co sites act as highly efficient catalytic centers and enhance CO_2_ adsorption through open Co porphyrin sites, enabling effective CO_2_ activation. Moreover, the presence of Co promotes directional energy migration within the MOF, significantly suppressing electron–hole recombination and providing long-lived electrons for the reduction of adsorbed CO_2_ molecules.^51^

One of the most common organic linkers, the porphyrin unit, has been regarded as a key active site in photoredox reactions. In the recent past, the Deng group determined that the photocatalytic activity increases as the distance between the interactive sites reduces and developed a formula that quantitatively explains this association.^125^ The porphyrin MOFs that were used as representatives in this case are MOF-525, PCN-221, PCN-222, PCN-223, PCN-224, and AlPMOF, which possess different intermolecular distances at the interactive sites. Nevertheless, excessive proximity of porphyrin active sites will decrease the size of the pore of MOFs, thereby inhibiting reaction kinetics. To overcome this, the authors developed a family of MOFs by creating vacancies by the partial removal of porphyrin linkers to form mesopores to favour the diffusion of the substrates. The results highlight the importance of the spatial arrangement of linker active sites, showing that shorter distances can theoretically maximize catalytic efficacy. Also, active sites on organic linkers could be introduced through postsynthetic modification. Pang *et al.* synthesized a mesoporous MOF, PCN-808, based on Zr_6_-oxo clusters and tetratopic carboxylate ligands. Using a linker installation procedure, the pore environment of PCN-808 was modified by precisely incorporating a linear ruthenium metalloligand [Ru(bpy)_3_]^2+^ (H_2_BDBR) into the framework, producing PCN-808-BDBR as an effective photocatalyst (Fig. 11b).^126^

The synthesis relies on a CO_2_-assisted exfoliation strategy in which supercritical CO_2_ acts as both a chemical modulator and a structural driving force. In bulk Ni-BDC, adjacent coordination layers are held together by hydrogen bonding between coordinated and lattice water molecules. When exposed to supercritical CO_2_, these water molecules are partially displaced and replaced by CO_2_, which chemically adsorbs onto coordinatively unsaturated Ni sites. This adsorption weakens the interlayer hydrogen bonds and induces effective delamination of the 3D framework into single-layer nanosheets (Fig. 11c). Simultaneously, the strong interaction between CO_2_ and Ni stabilizes the exposed metal centers, preserving the ultrathin layered structure, and results in 2D MOFs with a high surface area, abundant active sites, and enhanced accessibility for CO_2_ activation.^127^

### 3.6 Pore confinement strategy

MOFs are porous materials with well-defined and tunable pore structures. When the pore size matches the diameter of a metal complex ion, the precursor can be introduced during MOF growth, allowing the complex ion to be trapped within the MOF cages.^128^ Due to this size matching, at most one metal precursor can be confined within each pore, effectively separating the metal species at the atomic level.^129^ A subsequent mild pyrolysis step decomposes the precursor without damaging the overall framework of the MOF, while the resulting single metal atoms remain stabilized through interactions with the surrounding ligands. In this way, pore confinement acts as a pre-separation strategy that suppresses metal agglomeration during heat treatment by providing spatial isolation of the metal centers.^130^

**Fig. 12:**
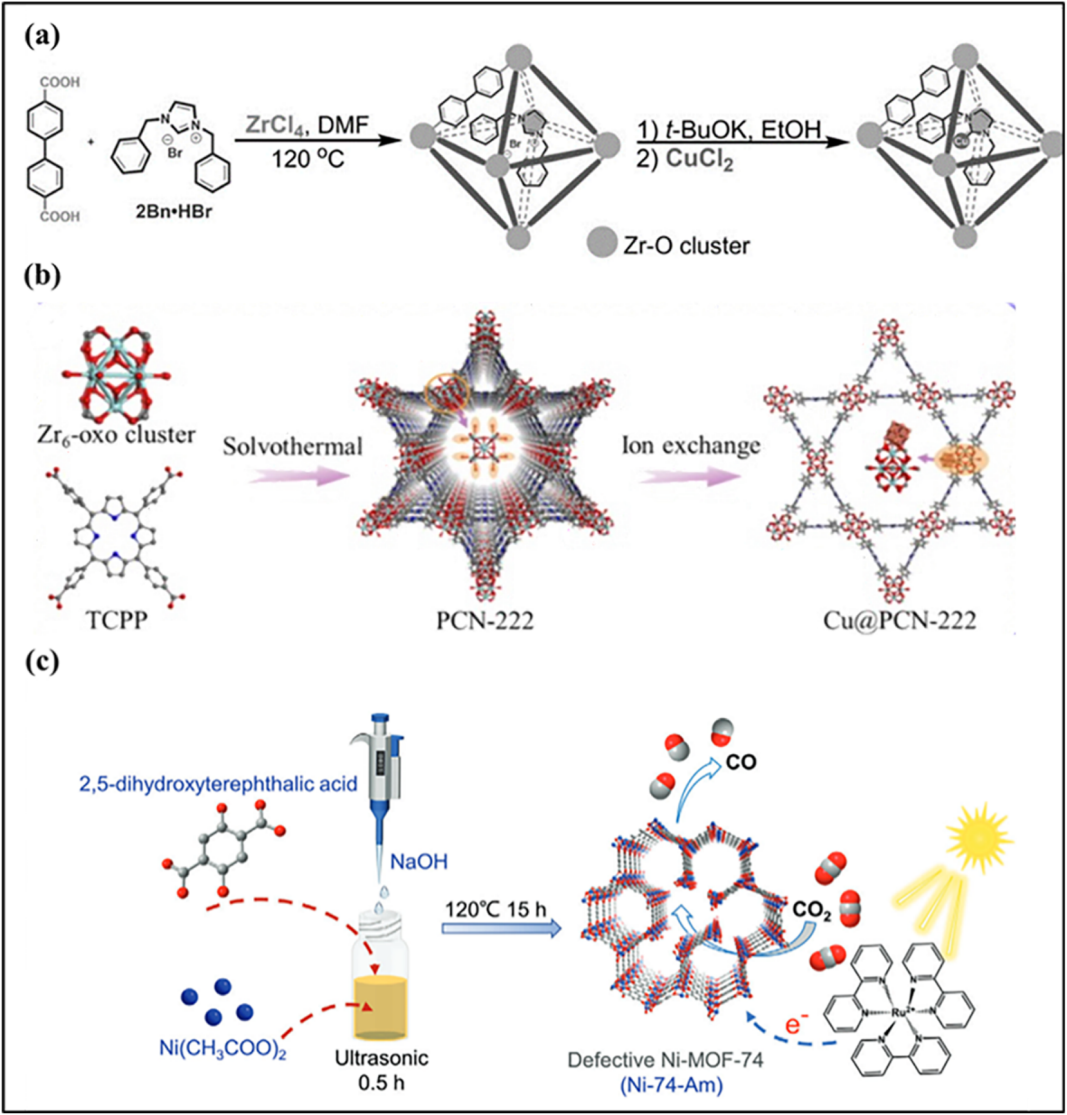
(a) Schematic of the preparation of SAC 2Bn-Cu@UiO-67. Reproduced with permission from ref. 131. (Copyright 2022 Wiley-VCH). (b) Schematic diagram of the fabrication of Cu/PCN-222. Reproduced with permission from ref. 132. (Copyright 2025 Elsevier). (c) Schematic diagram of the fabrication of Ni-74-Am. Reproduced with permission from ref. 133. (Copyright 2024 WILEY-VCH).

Shenghua Chen and coworkers reported a pore-confinement strategy in which a molecular *N*-heterocyclic carbene-ligated Cu complex was incorporated into an MOF during framework assembly. Because the size of the Cu-NHC complex matches the pore dimensions of the MOF, each cavity can host only one metal center, which ensures atomic-level separation and prevents Cu aggregation. The surrounding organic linkers and confined pore environment stabilize the Cu atoms through strong coordination and spatial restriction (Fig. 12a). This approach produces uniformly dispersed single-atom Cu sites embedded within the MOF, providing a well-defined and robust catalytic platform with high activity and stability.^131^ Bao *et al.* anchored Cu species onto open Zr_6_-oxo clusters of the Zr-based MOF PCN-222 *via* post-synthetic ion exchange, confining Cu within the MOF nanopores to generate strong metal-support interactions (SMSIs) (Fig. 12b). The resulting Cu@PCN-222 catalyst shows high CO_2_ hydrogenation activity toward methanol, achieving a space-time yield of 437.4 mg MeOH g_cat_^−1^ h^−1^ with good stability over 100 h. Detailed experimental and theoretical analyses reveal that Zr^4+^–O^2−^(–Cu^2+^) and Zr^4+^–Cu^2+^ interfacial sites act as highly efficient centers for CO_2_ and H_2_ adsorption and activation, while pore confinement modulates the electronic structure of Cu, favoring H_2_ activation and methanol selectivity.^132^

### 3.7 Defect engineering and vacancy control

**Fig. 13:**
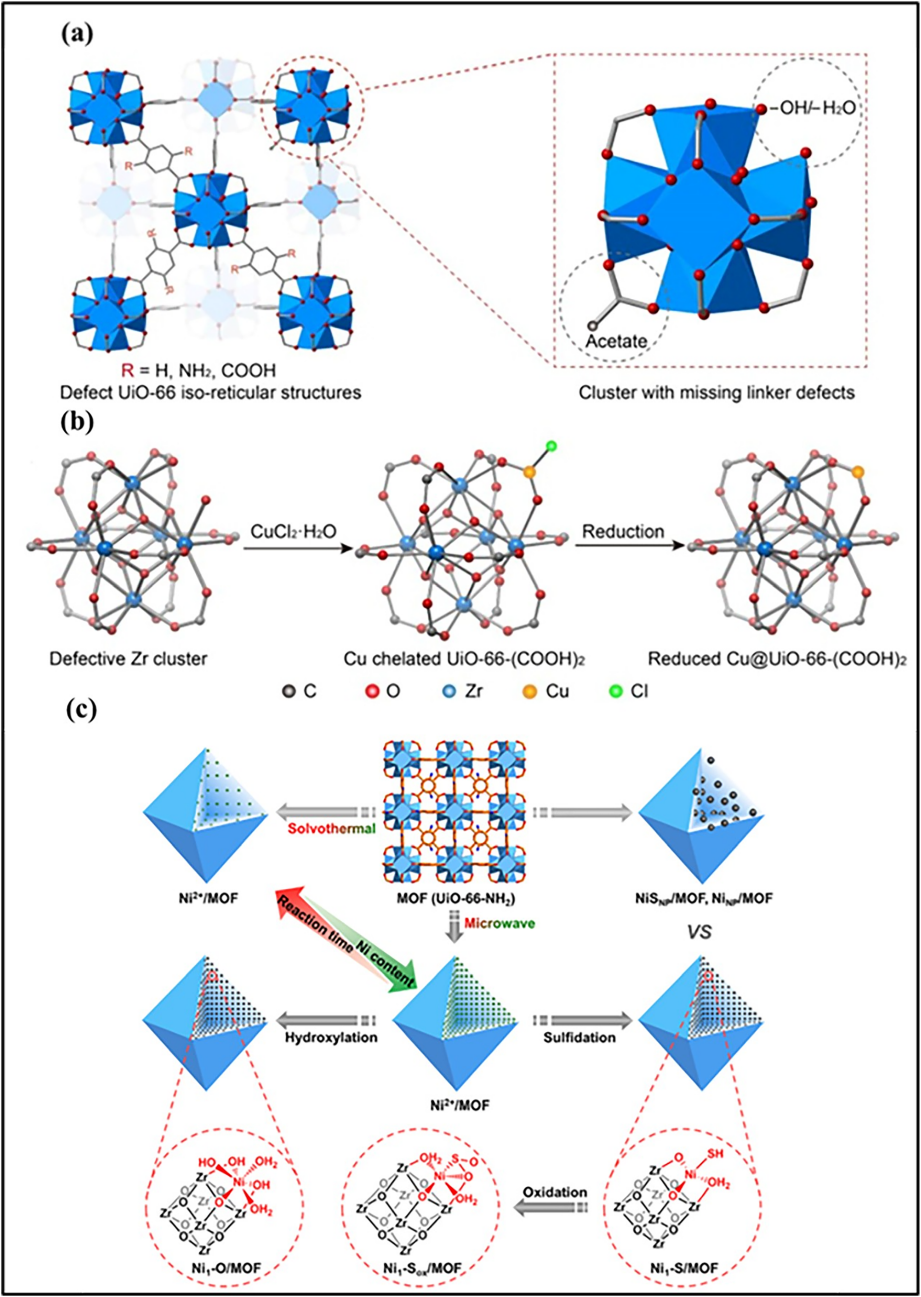
(a) Schematic structures of defective UiO-66 isoreticular MOFs with different functional groups, highlighting the Zr_6_ secondary building unit of UiO-66-(COOH)_2,_ where unsaturated metal sites are occupied by –OH/–H_2_O (H atoms are omitted for clarity). (b) Illustration of the synthesis of single-atom Cu@UiO-66-(COOH)_2_, in which Cu precursors coordinate to dangling oxygen species on defective Zr nodes and are subsequently reduced to form isolated Cu sites. Reproduced with permission from ref. 136. (Copyright 2023 Elsevier). (c) Scheme of showing Ni^2+^ decoration in UiO-66-NH_2_*via* an efficient microwave-assisted method. Reproduced with permission from ref. 137. (Copyright 2021 American Chemical Society).

Structural defects, including vacancies and interstitials, can adjust the coordination environment of metal centers and regulate their electronic properties. These defects provide additional active sites, enhance the stabilization of isolated metal atoms against aggregation, and modify the adsorption behavior of reactants, leading to improved catalytic performance.^134,135^ Yong-Li Dong *et al.* reported the construction of a defect-rich, amorphous Ni-MOF-74 (Ni-74-Am) by regulating synthesis temperature and solvent environment to induce ligand deficiencies (Fig. 12c). The resulting material contains abundant coordinatively unsaturated Ni sites and a mesoporous framework, which significantly improves photogenerated charge separation, carrier transport, and CO_2_ activation. Owing to these structural and electronic advantages, Ni-74-Am achieves a high CO evolution rate (1.38 mmol g^−1^ h^−1^) with 94% selectivity under visible-light irradiation, markedly outperforming crystalline Ni-MOF-74. This study highlights ligand-defect engineering as an effective strategy for enhancing MOF-based photocatalytic CO_2_ reduction.^133^ Wu *et al.* developed a Zr-based MOF-supported single-atom Cu catalyst (Cu@UiO-66-(COOH)_2_) by introducing controlled linker defects into the UiO-66 framework (Fig. 13a and b). The missing linkers create coordinatively unsaturated Zr_6_ nodes terminated with –OH/–H_2_O groups, which serve as strong anchoring sites for isolated Cu atoms and prevent their aggregation during synthesis and catalysis. Owing to the uniform atomic dispersion and strong metal-support interaction, the catalyst exhibits excellent activity and selectivity for the catalytic transfer hydrogenation of biomass-derived levulinic acid to γ-valerolactone. The high performance is attributed to the synergistic effect between Cu^+1^ single-atom sites responsible for catalytic activation and the acidic Zr_6_ nodes and carboxyl groups that promote intermediate transformation, resulting in high stability and recyclability.^136^

Xing Ma *et al.* reported a microwave-assisted strategy for the synthesis of high-loading SACs on Zr-based MOFs. Metal salts such as NiCl_2_·6H_2_O, CoCl_2_·6H_2_O, CuCl_2_·2H_2_O, and RuCl_3_ were dispersed with the MOF in acetonitrile and rapidly heated to 85 °C for 30 min under microwave irradiation. The –O/OH_*x*_ groups at defect sites of the Zr_6_-oxo clusters serve as anchoring points, stabilizing single metal atoms and preventing aggregation. Using this method, Ni loading reached 4.83 wt%, significantly higher than that in conventional solvothermal routes (1.01–1.37 wt%). Post-synthetic treatments, such as sulfidation or hydroxylation, allow modulation of the metal coordination environment, yielding atomically dispersed, highly accessible single-atom sites for photocatalysis^137^ (Fig. 13c).

Different incorporation strategies offer distinct benefits. Table 1 shows the overview of the main methods used to incorporate and stabilize single-atom catalysts (SACs) within MOFs. Anchoring single atoms at metal nodes provides high stability. Linker metallation allows control over electronic conductivity. Pore confinement suppresses aggregation during thermal treatment. However, these methods often suffer from low metal loading or limited scalability. Future work must address controllable high-density single-atom incorporation without sacrificing framework integrity or photocatalytic activity.

**Table 1:** MOF-based single-atom catalysts and incorporation strategies

MOF single atom system	MOF platform/support	Single atoms (SAs)	SA incorporation strategy	Ref.
Cu-UiO-66	UiO-66	Cu	Metal-node anchoring	108
Sn-ZIF-8	ZIF-8	Sn	Node ion exchange	109
Zn, N-MIL-125(Ti)	MIL-125(Ti)	Zn	*In situ* co-doping	110
M-Zr-MOFs	UiO-66, MOF-808, and NU-1000	Cu, Fe, Ni, and Au	Node photodeposition	111
Re-UiO-66	UiO-66-poly[bpy]	Re	Surface functionalization + postmetalation	112
Co, InCN	NH_2_-MIL-68 → CN	Co and In	MOF-derived pyrolysis	118
Co-MOF nanosheets	Co-MOF	Co	Ultrathin 2D MOF assembly	120
Fe-MOF/C–Ni NS	Fe-SACs/C–Ni NS	Fe	Pre-synthetic incorporation	123
Co-MOF-525	MOF-525	Co	Linker metallation	51
Co-PCN series	PCN-221-224	Co	Linker distance engineering	125
Ru-PCN-808	PCN-808	Ru	Postsynthetic linker installation	126
Ni-BDC nanosheets	Ni-BDC	Ni	CO_2_-assisted exfoliation	127
Cu-UiO-67	UiO-67	Cu	Nanopore confinement	131
Cu-PCN-222	PCN-222	Cu	Ion exchange + confinement	132
Ni-MOF-74-Am	Ni-MOF-74-Am	Ni	Defect engineering	133
Cu-UiO-66-(COOH)_2_	UiO-66-(COOH)_2_	Cu	Defect-assisted anchoring	136
M-UiO-66-NH_2_	UiO-66-NH_2_	Ni, Co, Cu, and Ru	Microwave-assisted anchoring	137

## 4 Characterization techniques of MOF-based SACs

The catalytic activity of SACs can only be fully understood through precise structural characterization of the isolated metal species. SACs differ fundamentally from conventional nanoparticle catalysts due to their atomic dispersion and unique electronic environments and therefore require advanced characterization techniques.^138^ A combination of spectroscopic, microscopic, and structural methods is commonly employed to elucidate their atomic structure, coordination environment, and electronic properties.^139^ Techniques such as X-ray absorption spectroscopy (XAS), including XANES and EXAFS, and X-ray photoelectron spectroscopy (XPS) provide valuable information on bonding states and local coordination.^106,140^ Meanwhile, isolated metal atoms can be directly visualized using high-resolution imaging techniques, particularly aberration-corrected HAADF-STEM and scanning tunneling microscopy (STM). These recent advances in electron microscopy, spectroscopy, and *in situ* techniques have significantly improved the understanding of the dynamic structural evolution of SACs under reaction conditions.^141^ Furthermore, density functional theory (DFT) calculations provide deeper insights into active-site geometry and reaction mechanisms, thereby enabling a more comprehensive understanding of their catalytic performance, particularly in the CO_2_ reduction reaction.^142^

### 4.1 X-ray absorption spectroscopy (XPS)

**Fig. 14:**
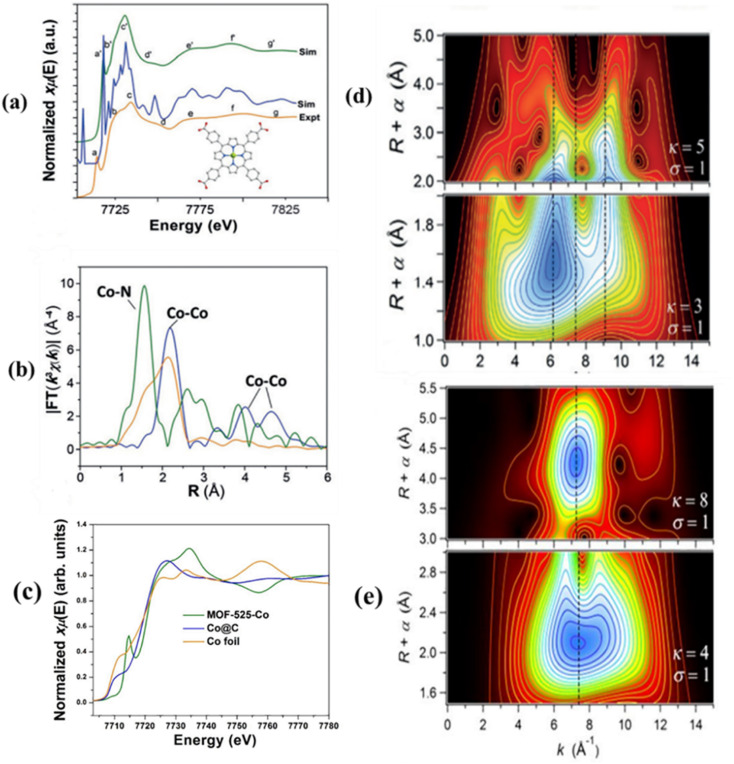
(a) Experimental Co K-edge XANES spectrum of MOF-525-Co (orange) compared with the corresponding theoretical spectrum; the non-convoluted calculated spectrum is shown for clarity. (b) Fourier-transformed magnitudes of Co K-edge EXAFS spectra (without phase correction) for Co foil, Co@C, and MOF-525-Co. (c) Normalized Co K-edge XANES spectra of MOF-525-Co and reference samples. (d) Wavelet transform of the *k*^3^-weighted Co K-edge EXAFS signal of MOF-525-Co, showing Co–N contributions and the absence of Co–Co scattering. (e) Wavelet transform of the *k*^3^-weighted Co K-edge EXAFS signal of Co foil, displaying characteristic Co–Co coordination features. Reproduced with permission from ref. 51. (Copyright 2016 Wiley-VCH).

High-energy X-rays generated at synchrotron radiation facilities provide powerful tools for probing materials and surface chemical processes at the atomic scale.^143^ Within XAS, X-ray absorption near-edge structure (XANES) and extended X-ray absorption fine structure (EXAFS) are widely employed to monitor oxidation states, coordination environments, and bond distances, allowing detailed insight into dynamic structural changes.^144,145^ The position and spectral features of the XANES region are closely associated with the oxidation state and local electronic structure of the absorbing atom, while variations in edge energy offer a reliable means to evaluate changes in metal valence.^140^ In contrast, EXAFS analysis yields quantitative information on the local coordination environment, including coordination numbers, neighboring atomic species, and interatomic bond lengths. Importantly, the absence of metal–metal scattering paths in EXAFS spectra serves as direct evidence for the formation of isolated single-atom sites.^146^ Together, XANES and EXAFS provide complementary and comprehensive structural and electronic information essential for understanding the nature and stability of single-atom catalysts. For instance, Co K-edge X-ray absorption spectroscopy was used to determine the coordination environment of Co single atoms in a MOF. EXAFS Fourier and wavelet transform analyses showed the absence of Co–Co scattering paths, confirming atomic dispersion of Co as isolated sites. EXAFS fitting indicated a Co–N_4_ coordination with a bond length of approximately 1.95 Å, consistent with a square-planar geometry and coordinatively unsaturated active sites. XANES spectra differed markedly from those of metallic Co, evidencing Co–N bonding, while the characteristic pre-edge feature arising from a 1s → 4p transition further supported the square-planar symmetry. The agreement between experimental and simulated XANES spectra validated the isolated nature and electronic structure of the Co single-atom sites (Fig. 14a–e).^51^

**Fig. 15:**
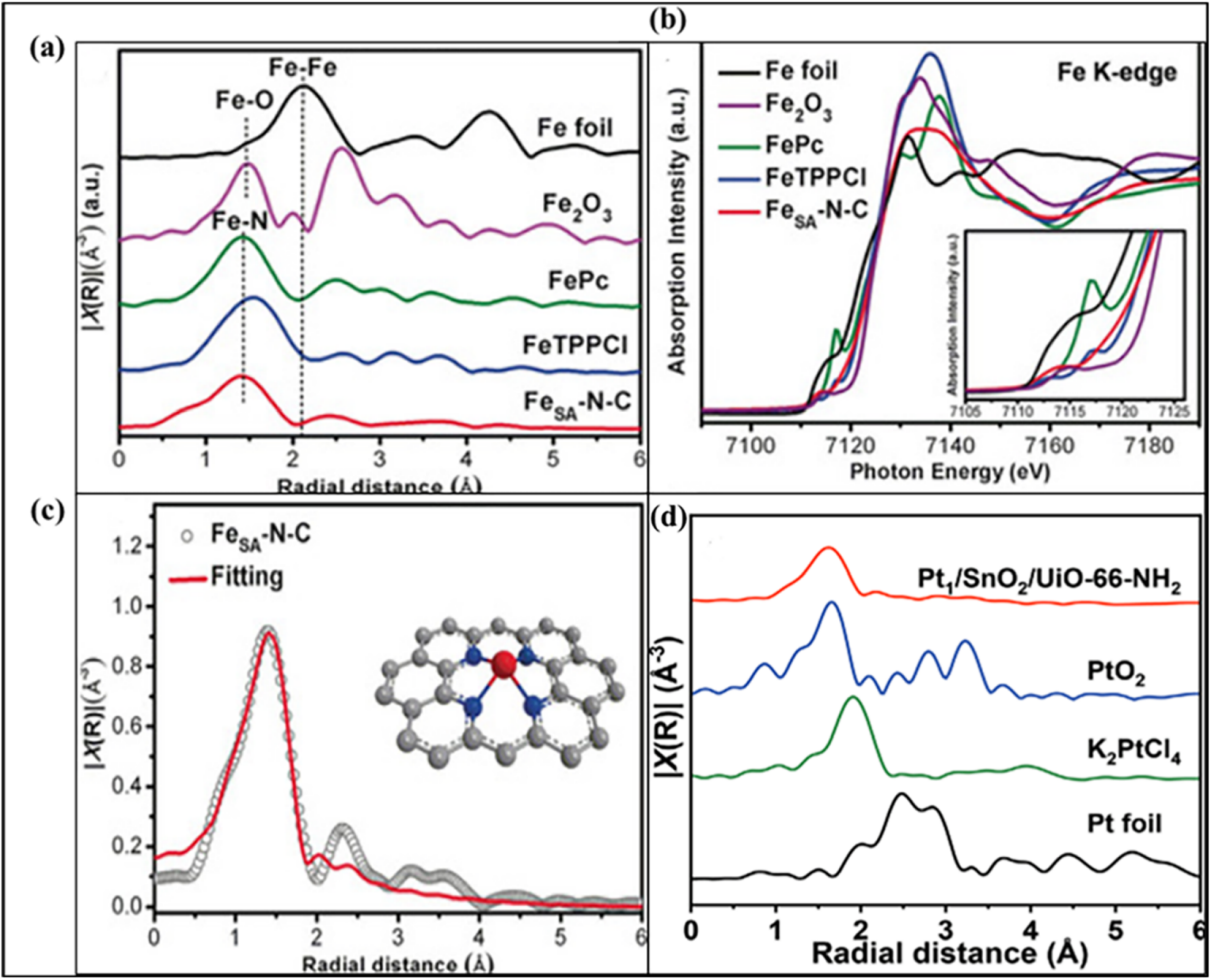
(a) Fe K-edge XANES spectra of Fe_SA_–N–C, FeTPPCl, FePc, Fe_2_O_3_, and Fe foil. (b) Fourier-transformed EXAFS (FT-EXAFS) spectra of the same samples. (c) EXAFS fitting for Fe_SA_–N–C (inset: structural model, Fe red, N blue, and C gray). Reproduced with permission from ref. 147. (Copyright 2018 WILEY-VCH). (d) FT *k*^2^-weighted *χ*(*k*) EXAFS spectra of Pt_1_/SnO_2_/UiO-66-NH_2_, PtO_2_, K_2_PtCl_4_, and Pt foil. Reproduced with permission from ref. 148. (Copyright 2022 WILEY-VCH).

Jiao *et al.* demonstrated that in a MOF-derived Fe_SA_–N–C catalyst, the Fe K-edge XANES spectrum shows the absorption threshold positioned between those of Fe_2_O_3_ and Fe foil, indicating positively charged Fe^*δ*+^ species stabilized by nitrogen coordination (Fig. 15a). EXAFS analysis further resolved the Fe–N scattering paths with an average bond distance of ∼1.44–1.95 Å and revealed no detectable Fe–Fe contributions at ∼2.13 Å, confirming the formation of atomically dispersed Fe sites (Fig. 15b). Wavelength transform analysis of the EXAFS signal also supported the absence of Fe–Fe interactions and highlighted the localized coordination environment of Fe atoms. These combined X-ray measurements provide direct evidence of isolated Fe–N_4_ single-atom sites and are widely employed to verify the formation, electronic structure, and stability of single-atom active centers in MOF-derived SACs (Fig. 15c).^147^ Jianfei Sui and coworkers determined that the Pt K-edge XANES spectra of Pt_1_/SnO_2_/UiO-66-NH_2_ indicate that the Pt single atoms carry a partial positive charge between 0 and +2. EXAFS analysis shows a prominent peak at ∼1.63 Å, corresponding to Pt–O bonds, while no Pt–Pt (∼2.49 Å) or Pt–Cl (∼1.9 Å) signals are observed. EXAFS fitting suggests that each Pt atom is coordinated by approximately four oxygen atoms (Fig. 15d).^148^

### 4.2 X-ray photoelectron spectroscopy (XPS)

**Fig. 16:**
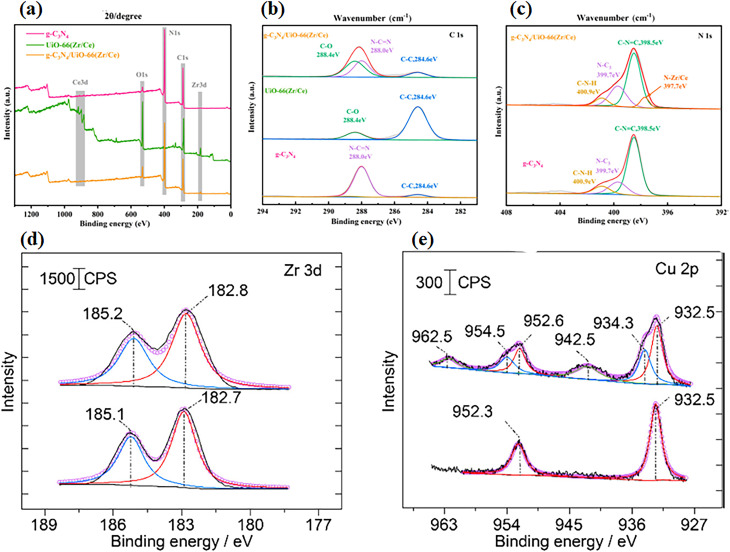
(a) Spectrum confirming the presence of C, N, O, Zr, and Ce; (b) high-resolution C 1s spectrum; (c) high-resolution N 1s spectrum. Reproduced with permission from ref. 150. (Copyright 2023 American Chemical Society). (d) Spectrum of Cu/UiO-66 (the Zr 3d region) and (e) Cu 2p region recorded after CO oxidation. Reproduced with permission from ref. 108. (Copyright American 2019 Chemical Society).

It is a powerful surface-sensitive technique for analyzing elemental composition and oxidation states in heterogeneous catalysts. Advanced *in situ* XPS measurements, particularly when combined with synchrotron radiation, provide deeper insight into the nature, stability, and evolution of active sites such as single atoms under working conditions.^137,149^ For example, Weiwei Wang *et al.* employed XPS to analyze UiO-66(Zr/Ce), a system that reveals characteristic peaks associated with metal oxygen coordination and ligand environment, confirming successful incorporation of active sites. Deconvolution of the C 1s spectrum reveals contributions from sp^2^-hybridized C–C bonds and C–O species associated with the UiO-66 framework. The N 1s spectrum can be resolved into multiple components corresponding to metal–nitrogen coordination (N–Zr/Ce), C–N=C, and sp^2^-hybridized N–H species. Such detailed spectral analysis underscores the capability of XPS to identify metal-support interactions and local electronic environments, making it a crucial tool for characterizing MOF-based and single-atom catalysts (Fig. 16a–c).^150^ Ali M. Abdel-Mageed *et al.* analyzed the chemical states of Cu species in fresh and spent Cu/UiO-66 catalysts. The Zr 3d spectra showed nearly identical binding energies for the fresh and used samples, indicating that the Zr^4+^ framework remained structurally stable during the reaction. In contrast, the Cu 2p_3/2_ peak shifted slightly to higher binding energy after CO oxidation, accompanied by the appearance of characteristic satellite peaks, confirming the partial oxidation of Cu species from Cu^0^/Cu^+^ to Cu^2+^ under reaction conditions. These results demonstrate the effectiveness of XPS in tracking oxidation state changes and metal-support stability in MOF-supported catalysts.^108^

### 4.3 Electron microscopy techniques

**Fig. 17:**
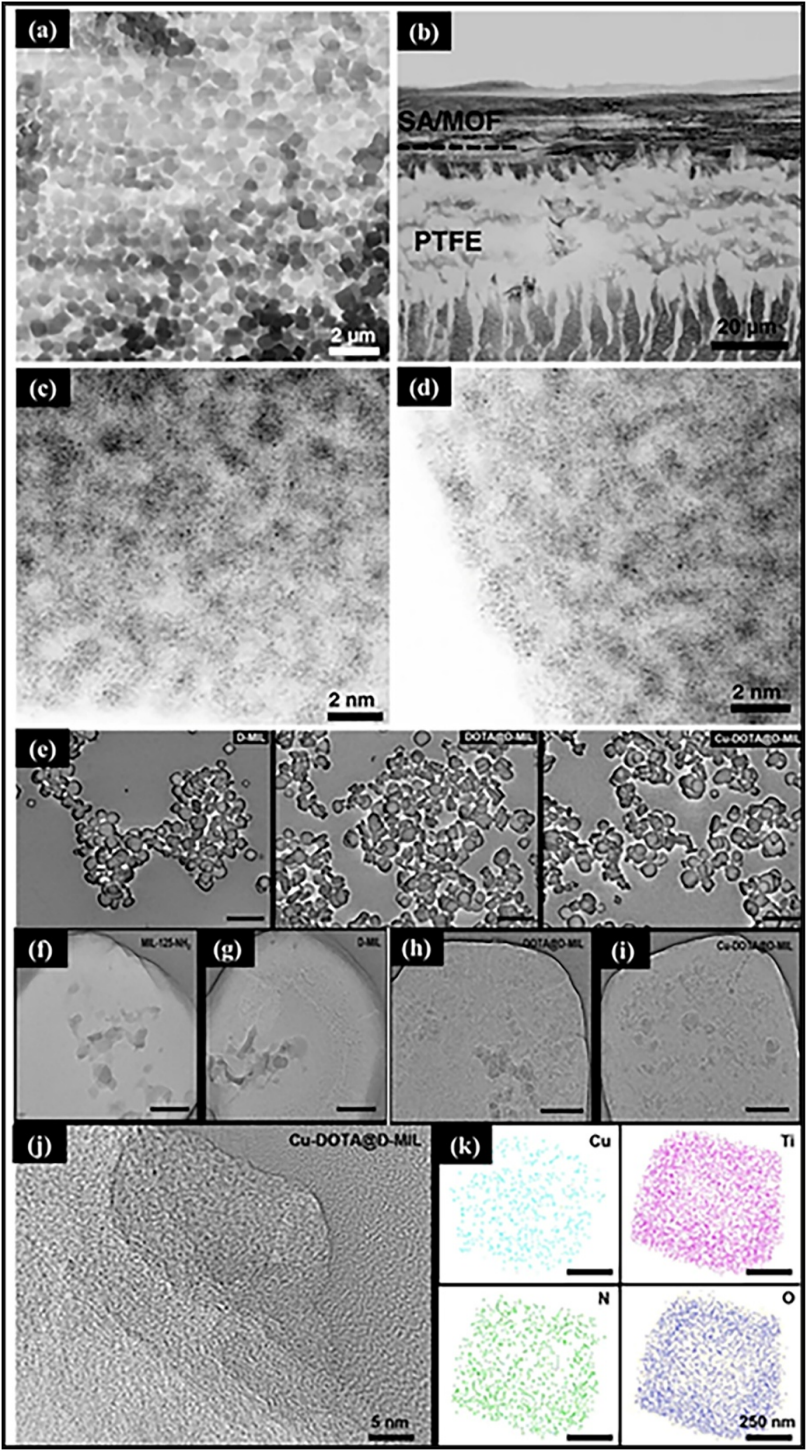
(a) SEM image of Ir_1_/A-aUiO particles. (b) Corresponding EDS mapping (c) and (d) Ir_1_/A-aUiO and Pd_1_/A-aUiO, indicating the atomic dispersion of metal species in A-aUiO matrices. Reproduced with permission from ref. 154. (Copyright 2021 Springer Nature). (e) SEM images of D-MIL, DOTA@D-MIL, and Cu-DOTA@D-MIL; scale bar: 5 µm. (f–i) TEM images of MIL-125-NH_2_, D-MIL, DOTA@D-MIL, and Cu-DOTA@D-MIL; the scale bar is 50 nm. (j and k) HRTEM and elemental mapping of Cu-DOTA@D-MIL. Reproduced with permission from ref. 155. (Copyright 2026 American Chemical Society).

Electron microscopy techniques such as SEM, TEM, and HAADF-STEM are widely used to examine the morphology, microstructure, and elemental distribution of catalysts.^151^ When combined with EDS, these methods enable nanoscale mapping of metal species within MOF structures. In particular, aberration-corrected HAADF-STEM enables the direct visualization of isolated metal atoms, as the image contrast scales with the atomic number (*Z*^2^).^152,153^ Yu-Chen Hao *et al.* investigated the morphology and atomic dispersion of Ir_1_/A-aUiO and Pd_1_/A-aUiO using SEM, TEM, HAADF-STEM, and aberration-corrected HAADF-STEM. SEM and TEM images show that the A-aUiO particles exhibit a polyhedral morphology with sizes around 100–200 nm, which is preserved after Ir and Pd incorporation (Fig. 17a and b). HAADF-STEM analysis reveals no formation of metal nanoparticles, while aberration-corrected HAADF-STEM images display isolated bright spots corresponding to atomically dispersed Ir and Pd species (Fig. 17c and d). Elemental mapping further confirms the uniform distribution of Zr, O, C, Ir, and Pd within the MOF matrix, demonstrating the successful formation of single-atom catalytic centers.^154^ Yan Che *et al.* reported the preparation of Cu-DOTA@D-MIL *via* defect engineering of MIL-125-NH_2_ and site-specific anchoring of DOTA, followed by Cu metalation. SEM and TEM images (Fig. 17e–i) show that the disk-like morphology is retained after modification, while HRTEM and elemental mapping (Fig. 17j and k) confirm atomically dispersed Cu without nanoparticle formation.^155^

**Fig. 18:**
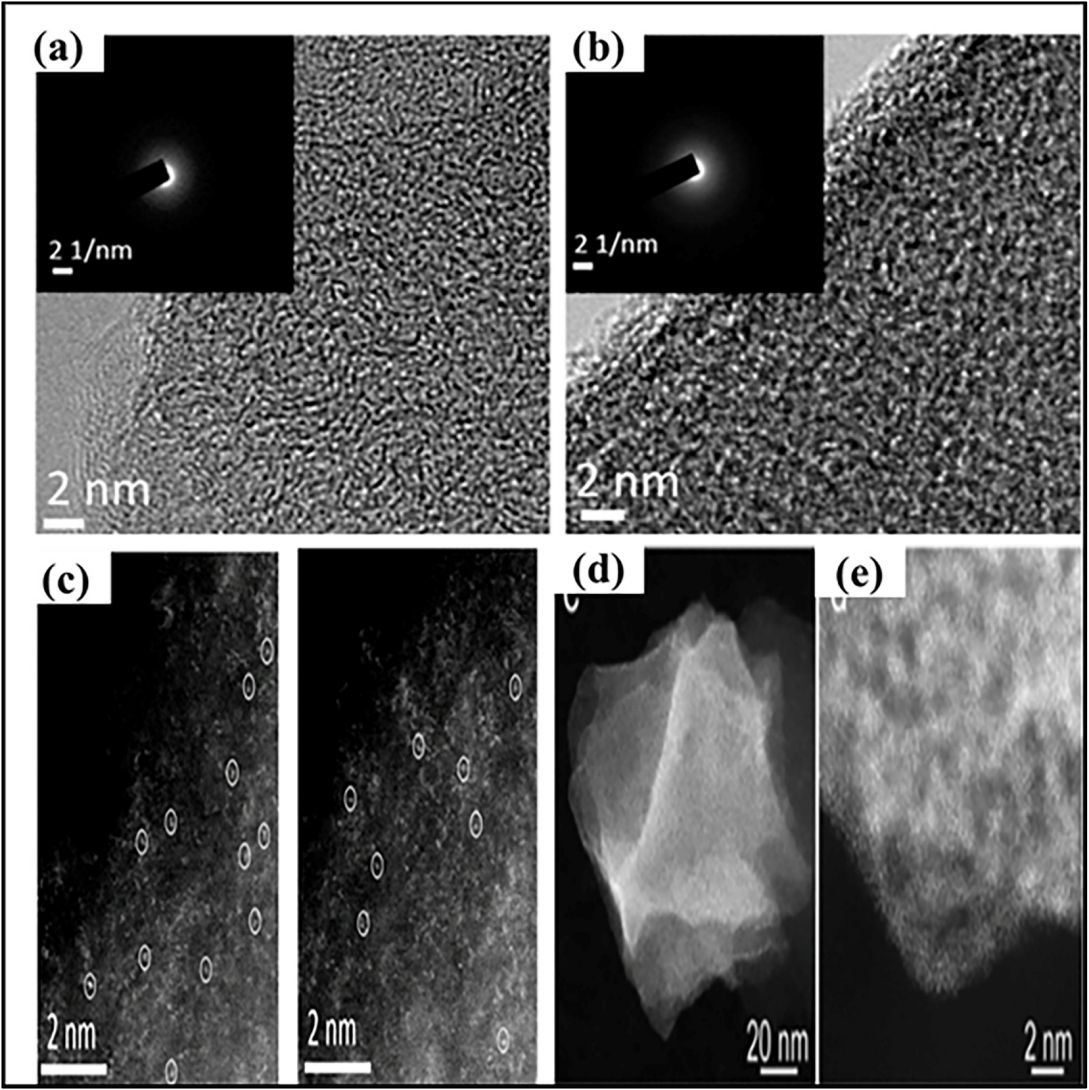
(a) and (b) HRTEM images (the inset image shows the corresponding SAED pattern) and (c) (AC-HAADF-STEM) images of Mn_SA_/NC and Mn_SA_/SNC.^156^ (Copyright 2021 American Chemical Society). (d and e) AC HAADF-STEM image. Reproduced with permission from ref. 131. (Copyright 2022 WILEY-VCH).

The Tan group reported the amorphous carbon structure of MnSA/NC and MnSA/SNC (Fig. 18a and b) without any observable lattice fringes, indicating the absence of crystalline manganese species. No Mn nanoparticles or clusters were detected in the TEM images, suggesting the lack of metal aggregation. To further verify the dispersion of Mn species, aberration-corrected high-angle annular dark-field scanning transmission electron microscopy (AC-HAADF-STEM) was employed. As shown in Fig. 18c, isolated bright spots are clearly observed and can be attributed to individual Mn atoms uniformly dispersed on the carbon matrix, confirming their atomic dispersion. In addition, the corresponding energy-dispersive X-ray spectroscopy (EDS) elemental mapping images reveal a homogeneous distribution of Mn along with C, N, and S (for MnSA/SNC) throughout the structure, further demonstrating the successful formation of Mn single-atom sites on the MOF-derived carbon support.^156^ In another study, Shenghua Chen *et al.* showed that HAADF-STEM images of 2Bn-Cu@UiO-67 exhibit an octahedral morphology with particle sizes around 60 nm, differing from that of 2Bn@UiO-67 due to variations in aggregation (Fig. 18d). The images also display numerous bright spots corresponding to atomically dispersed Cu and Zr sites, confirming the absence of Cu nanoparticles (Fig. 18e). EDX elemental mapping demonstrates a uniform distribution of Cu throughout the UiO-67 framework, indicating successful incorporation of Cu and its coexistence with Zr.^131^ Advanced spectroscopic and microscopic techniques provide strong evidence for the atomic dispersion and coordination environment of SA sites. However, most analyses are performed *ex situ*, offering limited insight into dynamic structural changes under operating conditions. *In situ* characterization is therefore essential for correlating the active-site structure with photocatalytic performance.

## 5 Applications of MOF-based SACs for photocatalytic CO_2_ reduction

Photocatalytic CO_2_ reduction requires catalysts with suitable band structures to enable efficient light absorption, charge separation, and surface redox reactions. However, limited CO_2_ adsorption and complex multi-step reaction pathways often result in low efficiency and poor product selectivity.^157^ Integrating SACs into MOFs offers an effective strategy to overcome these challenges. The strong interfacial interaction between MOFs and isolated metal atoms enhances charge separation, increases active site utilization, and suppresses charge recombination.^158^ Consequently, MOF@SACs have emerged as promising platforms for improving photocatalytic efficiency and selectivity in CO_2_ reduction. Metalloporphyrin-based MOFs with atomically dispersed metal centers provide valuable model systems for understanding structure–activity relationships in photocatalytic CO_2_ reduction.

**Fig. 19:**
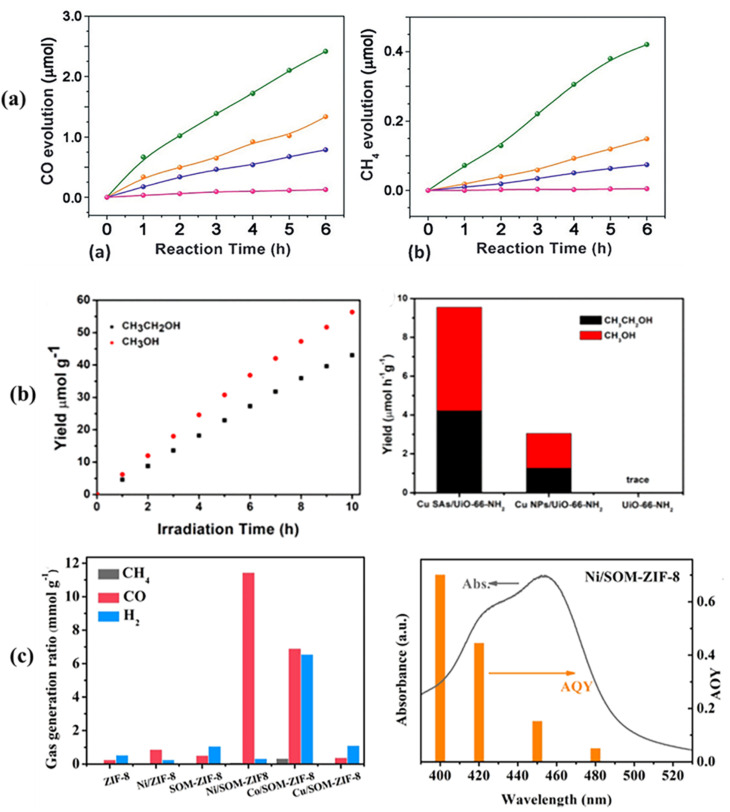
(a) Time-dependent evolution of CO and CH_4_ over MOF-525-Co (green), MOF-525-Zn (orange), and MOF-525 (purple) photocatalysts, and the H_6_TCPP ligand (pink). Reproduced with permission from ref. 51. (Copyright 2016 WILEY-VCH). (b) Production rate of liquid fuels of Cu SAs/UiO-66-NH_2_ and comparative yield rates of C_2_H_5_OH and CH_3_OH formation using different catalysts. Reproduced with permission from ref. 159. (Copyright 2020 American Chemical Society). (c) Photocatalytic CO_2_ reduction performance of ZIF-8, Ni/ZIF-8, SOM-ZIF-8, Ni/SOM-ZIF-8, Co/SOM-ZIF-8, and Cu/SOM-ZIF-8; AQY and UV-vis absorption spectrum of Ni/SOM-ZIF-8. Reproduced with permission from ref. 160. (Copyright 2023 American Chemical Society).

Co SAs embedded in porphyrin-based MOFs, such as MOF-525-Co, illustrate how the coordination environment affects photocatalytic performance. The coordinatively unsaturated Co sites maximize metal utilization and expose accessible active centers, enhancing CO_2_ adsorption and activation. Consequently, MOF-525-Co delivers CO and CH_4_ production rates of 200.6 and 36.7 µmol g^−1^ h^−1^, respectively, representing 3.13- and 1.93-fold improvements compared with pristine MOF-525 (Fig. 19a). The superior activity is attributed to the strong CO_2_ binding of isolated Co atoms and efficient charge transfer through the porphyrin framework.^51^ The role of the metal type and coordination environment is further highlighted by Cu and Ni single atoms in MOFs. Cu single atoms anchored onto UiO-66-NH_2_*via* amino groups using a photoinduced deposition method show enhanced charge separation and increased electron density at the Cu sites, which favors multi-electron CO_2_ reduction. This structural design results in CH_3_OH and C_2_H_5_OH production rates of 50.33 and 40.22 µmol g^−1^ h^−1^, respectively, significantly higher than those of their nanoparticle-based counterparts^159^ (Fig. 19b). Similarly, Ni single atoms incorporated into a hierarchically porous ZIF-8 framework (Ni/SOM-ZIF-8) illustrate the importance of combining atomic dispersion with macro-microporosity. The ordered porosity facilitates CO_2_ diffusion and adsorption, while isolated Ni atoms optimize reaction energetics. This results in a CO production rate of 4.2 mmol g^−1^ h^−1^ with 94% electron selectivity, roughly 47 times higher than that of pristine ZIF-8, and an apparent quantum yield of 0.71% under 400 nm irradiation (Fig. 19c).^160^

**Fig. 20:**
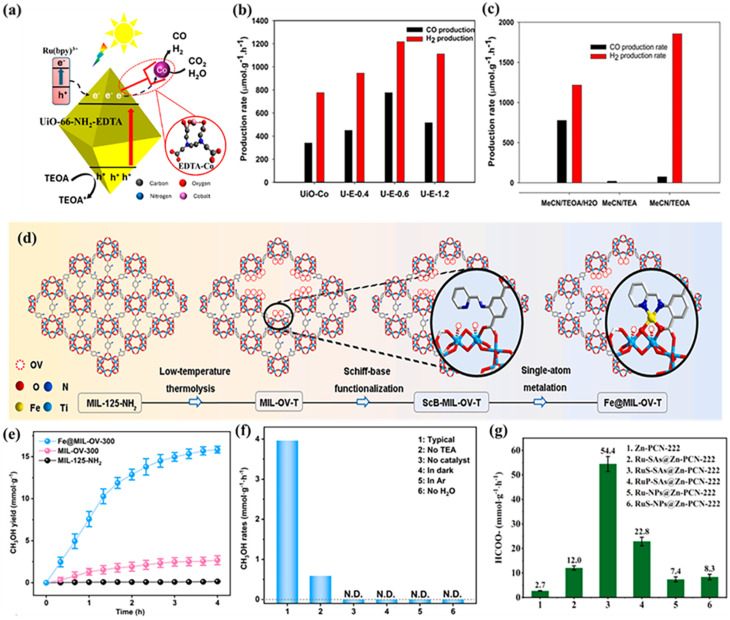
(a) Schematic illustration of the charge-transfer mechanism in UiO-66-NH_2_-EDTA-Co during photocatalytic CO_2_ reduction to syngas under light irradiation. (b) Gas production rates of UiO-66-NH_2_-Co and the samples U-E-0.4, U-E-0.6, and U-E-1.2. (c) Control experiments for the composite sample U-E-0.6 using different solvent systems (MeCN/TEOA/H_2_O, MeCN/TEOA, and MeCN/TEA). Reproduced with permission from ref. 161. (Copyright 2024 American Chemical Society). (d) Schematic illustration of the synthesis of Fe@MIL-OV-T. (e) Photocatalytic CH_3_OH yields over MIL-125-NH_2_, MIL-OV-300, and Fe@MIL-OV-300. (f) CH_3_OH generation rates of Fe@MIL-OV-300 under different reaction conditions. Reproduced with permission from ref. 162. (Copyright 2025 Elsevier). (g) Photocatalytic performances of the RuS-SAs@Zn-PCN-222 series under standard conditions: catalyst (3 mg), NH_3_BH_3_ (10 mg), methanol (5 mL), light irradiation (*λ* > 420 nm), reaction time (0.5 h). Reproduced with permission from ref. 163. (Copyright 2025 Elsevier).

The UiO-66-NH_2_-Co/EDTA photocatalyst highlights the impact of metal coordination and chelation on CO_2_ reduction. Co SAs are uniformly incorporated into the UiO-66-NH_2_ framework using EDTA as a chelating agent, creating accessible active sites that facilitate CO_2_ adsorption, activation, and conversion to syngas. This design achieves CO and H_2_ generation rates of 776.40 and 1217.29 µmol g^−1^ h^−1^, nearly double those of EDTA-free UiO-66-NH_2_-Co, with a CO/H_2_ ratio of about 1 : 2, making it well-suited for industrial syngas applications (Fig. 20a–c).^161^ In the case of Fe@MIL-OV-T, isolated Fe sites are introduced into a MIL-125-NH_2_-based framework through Schiff-base functionalization in an oxygen-vacancy-rich structure. This combination of defect engineering and atomic dispersion promotes efficient CO_2_ photoreduction, achieving a CH_3_OH production rate of 3.96 mmol g^−1^ h^−1^ with 98% selectivity under visible light. Even without a sacrificial agent, the catalyst retains a CH_3_OH production rate of 0.59 mmol g^−1^ h^−1^, demonstrating its intrinsic efficiency (Fig. 20d–f).^162^

**Fig. 21:**
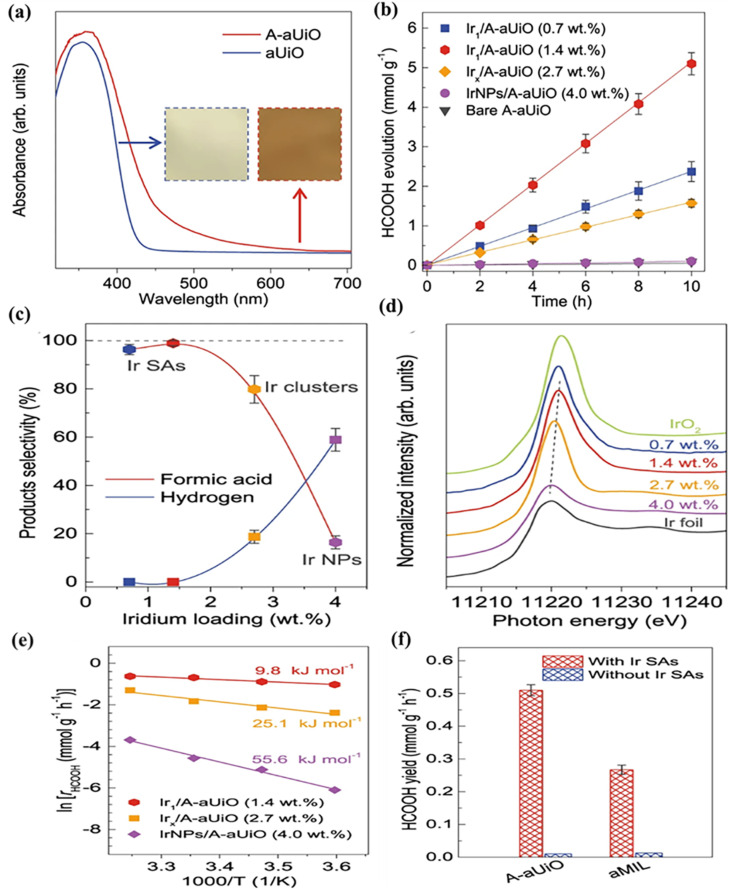
(a) UV-Vis diffuse reflectance spectra showing enhanced light absorption of defect-engineered A-aUiO. (b) HCOOH evolution over time for A-aUiO, Ir_1_/A-aUiO, Ir_*x*_/A-aUiO, and IrNPs/A-aUiO catalysts. (c) HCOOH and H_2_ selectivity as a function of Ir loading in Ir/A-aUiO catalysts. (d) Ir L-edge XANES spectra of Ir/A-aUiO with different Ir loadings; Ir foil and IrO_2_ are shown as references. The shift of the first XANES peak indicates decreasing metallicity with lower Ir loading. (e) Apparent activation energies (*E*_app_) for HCOOH formation over different catalysts. (f) HCOOH yields on A-aUiO and aMIL with and without Ir single atoms. Reproduced with permission from ref. 164. (Copyright 2021 Springer Nature).

Ru SAs immobilized on Zr_6_O_8_ clusters in the RuS-SAs@Zn-PCN-222 MOF further illustrate the role of metal-MOF electronic interactions. Stabilized by thiol (–SH) groups, the Ru sites enhance electron transfer and CO_2_ adsorption, promoting efficient coupling with surface H* species. This results in a formate (HCOO–) production rate of 54.4 mmol g^−1^ h^−1^, nearly 100% selectivity, and a turnover frequency of 440 h (Fig. 20g).^163^ Defect-engineered NH_2_-UiO-66 (A-aUiO) with isolated Ir single atoms at missing-linker sites shows that the combination of atomic dispersion and framework defects can dramatically improve activity and selectivity. Under visible-light irradiation (*λ* > 420 nm), Ir_1_/A-aUiO produces HCOOH at 0.51 mmol g^−1^ h^−1^ with 98–100% selectivity, while Ir clusters and nanoparticles produce more H_2_ and CO (Fig. 21a–c). The positively charged Ir sites (+3 to +4) reduce activation energy (9.8 kJ mol^−1^, Fig. 21e) and suppress side reactions. Incorporating Ir_1_/A-aUiO into a gas-permeable MOF membrane using a gas-membrane-gas (GMG) setup increases the formate yield to 3.38 mmol g^−1^ h^−1^, over six times higher than that of the particle-in-solution configuration, while retaining high selectivity and stability (Fig. 21f). Similar results are observed for Ir_1_/aMIL, demonstrating the synergy between MOF matrices and SACs in selective CO_2_ photoreduction.^164^

### 5.1 MOF-based SA nanocomposites

Despite the atomically dispersed catalytic sites and adjustable coordination spheres of MOF single-atom catalysts, their photocatalytic activities are fraught with challenges such as low electrical conductivity, limited photo-absorption ranges, rapid charge recombination rates, and instability.^39^ These challenges are effectively overcome by hybrid MOF-based composite systems and conductive supports, which help in improving photo-absorption ranges, facilitating charge separation and transportation, enhancing CO_2_ adsorption and activation abilities, and improving structural stability.^165^ The composite synergistic effect also promotes the control of reaction routes and the modulation of electronic structures for enhanced activities and stability. The strategy of making composites is crucial for fully exploring the potential of single-atom photocatalysts based on MOFs for solar-driven CO_2_ reduction.^166^

**Fig. 22:**
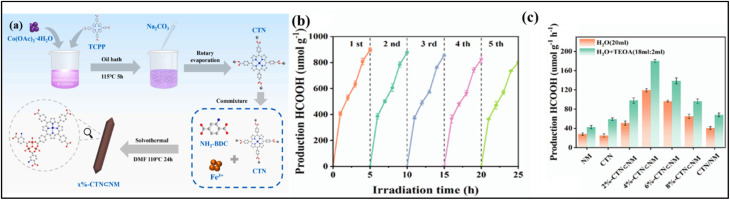
(a) Schematic representation for producing CTN ⊂ NM MOF structures *via* the one-pot method. (b) HCOOH generation rates of NM, CTN, CTN/NM, and CTN ⊂ NM in different systems. (c) Cyclic catalytic experiment with 4%-CTN ⊂ NM. Reproduced with permission from ref. 167. (Copyright 2026 Elsevier).

X. Li *et al.* synthesized a hybrid Co-metalloporphyrin (CoTCPPNa_4_) ⊂ NH_2_-MIL-101(Fe) (CTN ⊂ NM) *via* a one-pot approach. The optimized 4%-CTN ⊂ NM composite (Co/Fe = 0.40) achieves a HCOOH production rate of 119.11 µmol g^−1^ h^−1^ without sacrificial agents, a 4.2-fold enhancement over the pristine MOF (Fig. 22a–c). With sacrificial agents, the rate increases to 179.87 µmol g^−1^ h^−1^, while the catalyst retains its activity over multiple cycles, confirming excellent stability and structural integrity.^167^Table 2 shows the summary of recently reported MOF@SA photocatalysts for CO_2_ reduction. Beyond the photocatalytic reduction of CO_2_ to CO, the generated CO can be directly utilized in downstream organic transformations to produce high-value compounds. Monticelli *et al.* demonstrated that rapid and complete CO_2_ to CO conversion (<10 min, >80% yield) enables immediate application of CO in various carbonylation reactions, including the synthesis of amides, esters, ketones, aldehydes, carboxylic acids, and ynones. The study further extended this strategy to isotopically labeled CO ([^13^C], [^14^C], [^11^C]), allowing the straightforward preparation of radiolabeled pharmaceuticals with high isotopic purity and good radiochemical yields. These findings highlight the practical relevance of CO_2_ photoreduction for integrated catalytic systems and emphasize the importance of achieving high CO_2_ to CO conversion to maximize the efficiency of subsequent chemical transformation.^168^ Similarly, X. L. Ma *et al.* determined that the integration of CO_2_ photoreduction and subsequent carbonylation in a continuous tandem photocatalytic system highlights a practical route for sustainable chemical synthesis. By encapsulating CdS QDs within PCN-Co, the photogenerated electrons are efficiently transferred to Co sites, reducing CO_2_ to CO, while the holes oxidize alcohol substrates to generate α-hydroxybenzyl radicals that undergo C–C coupling. The produced CO can be immediately utilized in downstream carbonylation reactions catalyzed by Pd/PCN-Zn, enabling the gram-scale synthesis of high-purity amides and pinacols. This dual functionality addresses key challenges in solar-driven CO_2_ reduction, including the efficient utilization of photogenerated holes, high selectivity toward valuable products, and the integration of oxidation and reduction processes within a single, continuous-flow system. These findings demonstrate the feasibility of combining CO_2_ valorization with fine chemical synthesis, paving the way for practical applications of photochemical CO_2_ conversion.^169^

**Table 2:** Summary of recently reported MOF@SA photocatalysts for photocatalytic CO_2_ reduction

Photocatalyst	Single-atom	Light source	Product	Photocatalytic activity	Ref.
MOF-525-Co	Co	300 W Xe lamp (400 nm < *λ* < 800 nm)	CO and CH_4_	CO (200.6 µmol g^−1^ h^−1^)	51
				CH_4_ (36.7 µmol g^−1^ h^−1^)	
Cu-SAs/UiO-66-NH_2_	Cu	300 W Xe lamp (*λ* > 400 nm)	CH_3_OH and C_2_H_5_OH	CH_3_OH (5.33 µmol g^−1^ h^−1^)	159
				C_2_H_5_OH (4.22 µmol g^−1^ h^−1^)	
Ni/SOM-ZIF-8	Ni	300 W Xe lamp (*λ* > 400 nm)	CO	CO (4.2 mmol g^−1^ h^−1^)	160
UiO-66/EDTA-Co	Co	100 W Xe lamp	CO and H_2_	CO (776.4 µmol g^−1^ h^−1^)	161
				H_2_ (1217.29 µmol g^−1^ h^−1^)	
Fe@MIL-OV-T	Fe	300 W Xe lamp (420 nm < *λ* < 800 nm)	CH_3_OH	CH_3_OH (3.96 mmol g^−1^ h^−1^)	162
RuS-SAs@Zn-PCN-222	Ru	300 W Xe lamp (*λ* > 420 nm)	HCOO^−^	HCOO^−^ (54.4 mmol g^−1^ h^−1^)	163
CTN ⊂ NM	Co	300 W Xe lamp (*λ* > 420 nm)	HCOOH	179.87 (µmol g^−1^ h^−1^)	167

### 5.2 COF-based single-atom photocatalysts

**Fig. 23:**
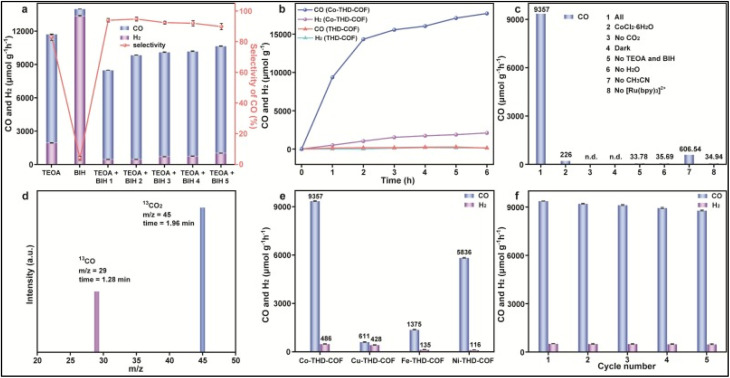
Photocatalytic CO_2_ reduction by Co-THD-COF: (a) CO and H_2_ production rates and CO selectivity with different sacrificial agents; (b) time-resolved CO_2_RR performance of THD-COF and Co-THD-COF; (c) CO_2_ photoreduction under various control conditions; (d) GC-MS spectra from ^13^CO_2_ reduction; (e) CO and H_2_ production on M-THD-COFs (M = Co, Cu, Fe, Ni); (f) CO and H_2_ generation over five consecutive cycles (error bars from three replicates) Reproduced with permission from ref. 170. (Copyright 2024 Elsevier).

**Fig. 24:**
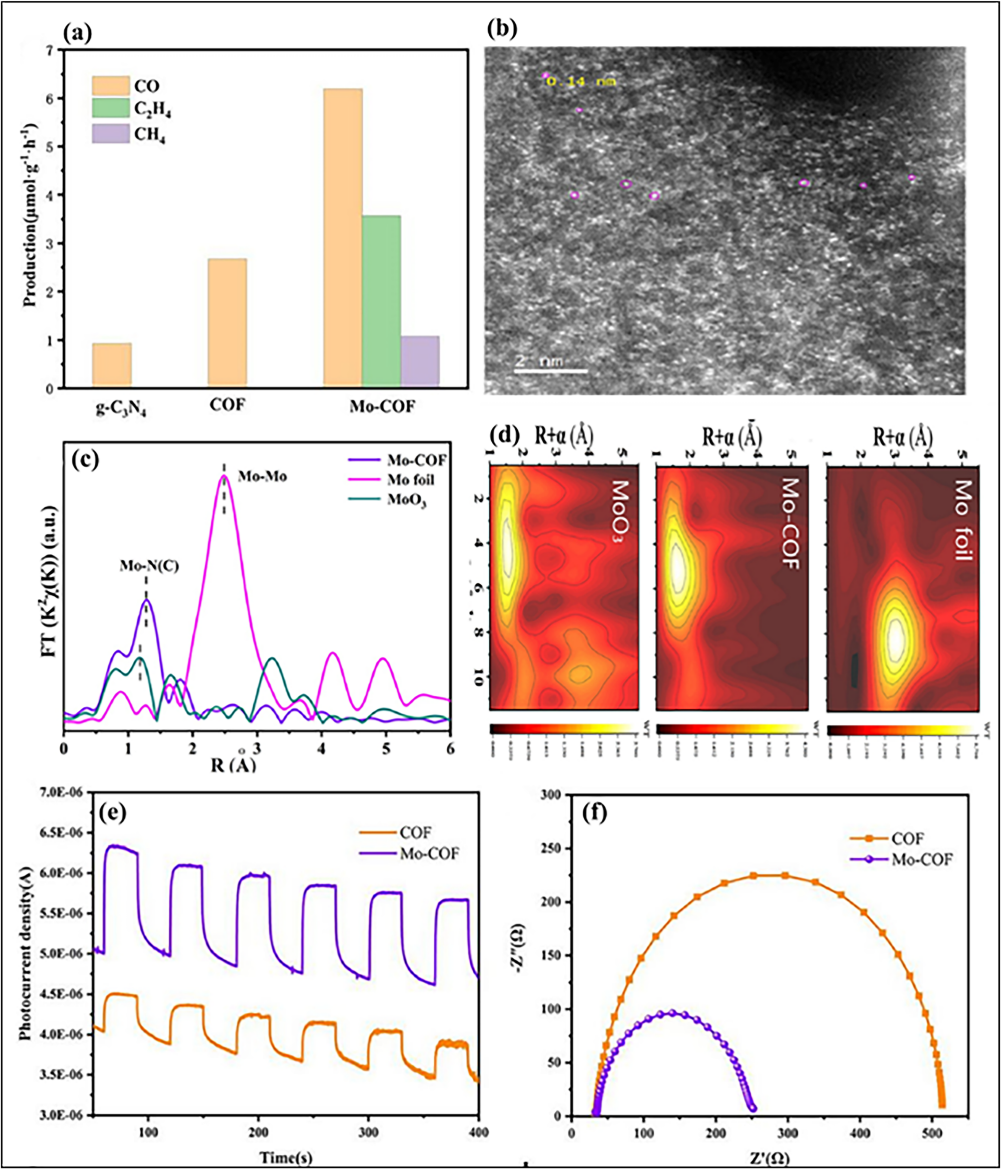
(a) Time courses of photocatalytic product formation. (b) Aberration-corrected HAADF-STEM image of Mo-COF showing single-atom Mo sites. (c) EXAFS analysis of Mo-COF (e); (d) Wavelet transform (WT) analysis of Mo foil, Mo-CO, and MoO_3_. (e) Transient photocurrent response and (f) electrochemical impedance spectra (EIS) of the COF and Mo-COF under visible light irradiation (*λ* ≥ 420 nm). Reproduced with permission from ref. 171. (Copyright 2021 Elsevier).

Covalent organic frameworks (COFs) provide an excellent platform for stabilizing SACs, allowing precise control over the coordination environment of metal centers. For instance, Pan *et al.* designed Co-THD-COF with bioinspired N, S-coordination sites that anchored single Co atoms, facilitating CO_2_ adsorption, improving charge separation, and lowering the energy barrier for *COOH intermediate formation. This system achieved a remarkable CO generation rate of 9357 µmol g^−1^ h^−1^ with 95.1% selectivity, even in natural seawater, demonstrating the potential of COF-based SACs as highly efficient photocatalysts (Fig. 23a–f).^170^ COFs with SA active sites, such as the MoN_2_-modified COF (Mo-COF), show significantly enhanced photocatalytic CO_2_ reduction performance. The Mo-COF produced C_2_H_4_ at a rate of 3.57 µmol g^−1^ h^−1^ with 32.92% selectivity, along with CH_4_ (1.08 µmol g^−1^ h^−1^) and CO (6.19 µmol g^−1^ h^−1^) under visible light (Fig. 24a). Structural characterization confirmed SA-Mo incorporation without cluster formation *via* HAADF-STEM (Fig. 24b) and EXAFS (Fig. 24c and d). Photoelectrochemical measurements revealed improved charge separation and electron transfer in Mo-COF, evidenced by higher photocurrent response and smaller Nyquist arc radius compared to the pristine COF (Fig. 24e and f).^171^

**Fig. 25:**
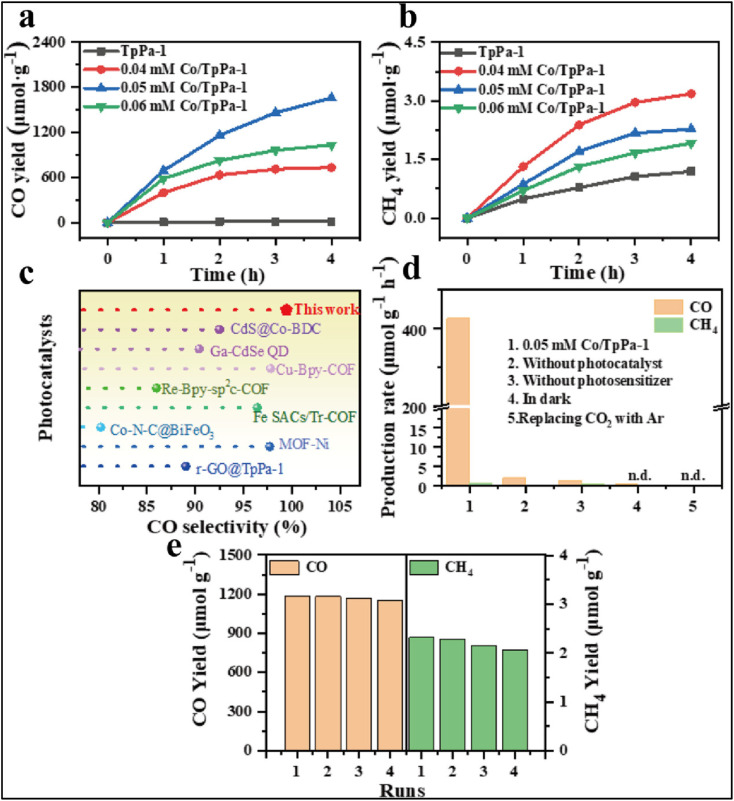
Photocatalytic CO_2_ reduction performance under visible light irradiation for TpPa-1 COFs and Co/TpPa-1 composites at different concentrations: (a) CO yield, (b) CH_4_ yield, (c) comparison of CO selectivity among SAC photocatalysts, (d) production rate after 3 h of visible light exposure, and (e) cycling stability test of the 0.05 mM Co/TpPa-1 composite. Reproduced with permission from ref. 172. (Copyright 2025 American Chemical Society).

Covalent organic frameworks (COFs) with anchored SA cobalt sites demonstrate excellent potential for photocatalytic CO_2_ reduction. The *in situ* coordination of Co with interlayer nitrogen atoms in keto-enamine TpPa-1 COFs ensures uniform dispersion of SAs, enhancing visible light absorption and charge separation. This design significantly increases CO production to 414.5 µmol g^−1^ h^−1^ with 99.45% selectivity (Fig. 25a–e), two orders of magnitude higher than that of pristine COFs, while retaining stability over multiple cycles. The formation of *COOH intermediates during the reaction confirms the single-atom Co sites as active centers, providing high efficiency and selectivity in CO_2_ reduction.^172^

## 6 Future directions

MOF-based single-atom photocatalysts have shown considerable potential for solar-driven CO_2_ conversion, yet several challenges remain. Establishing clear structure–activity relationships is essential, particularly by linking single-atom coordination environments with charge-transfer behavior and product selectivity. Precise control over metal-framework electronic interactions will be critical for further performance optimization. Limited solar-light utilization continues to restrict overall efficiency. Future designs should focus on extending light absorption while suppressing charge recombination through rational linker engineering and interfacial charge-transfer modulation, without compromising structural stability. Long-term durability and scalability also require greater attention, as framework degradation, metal migration, and limited single-atom loading hinder practical application. Developing robust MOF architectures that support high single-atom densities under prolonged irradiation is therefore necessary. Finally, *in situ* and *operando* characterization should be more widely employed to track dynamic changes in active sites and reaction intermediates. Such insights, combined with theoretical modeling, will guide the rational design of next-generation MOF-based single-atom photocatalysts for efficient and sustainable CO_2_ conversion.

## 7 Conclusion

MOF-based single-atom photocatalysts have emerged as a highly promising class of materials for solar-driven CO_2_ reduction, offering unique advantages over conventional nanoparticle systems. By confining isolated metal atoms within precisely defined coordination environments, MOF platforms enable near-complete metal utilization, enhanced exposure of active sites, and fine regulation of electronic structures. These features collectively lead to improved light harvesting, more efficient charge separation, and superior control over reaction intermediates and product selectivity. While MOF-based single-atom photocatalysts have demonstrated superior activity, stability, and selectivity, covalent organic frameworks (COFs) provide an additional platform with tunable porosity and electronic properties for stabilizing single-atom sites, offering further opportunities to enhance photocatalytic CO_2_ conversion. Throughout this review, it is evident that MOF-supported single-atom catalysts consistently outperform their nanoparticle and cluster counterparts in terms of activity, selectivity, and stability. For example, node-anchored and linker-coordinated SA systems frequently exhibit multi-fold enhancements in CO, CH_4_, CH_3_OH, or HCOO^−^ production rates, while simultaneously suppressing competing hydrogen evolution reactions. Comparative analysis across reported systems clearly demonstrates that atomically dispersed metal centers facilitate optimized adsorption energies for key intermediates such as *COOH, *CO, and *OCHO, thereby lowering reaction energy barriers and steering reaction pathways toward desired C_1_ or C_2_^+^ products.

In contrast to nanoparticle catalysts, which often suffer from metal aggregation, heterogeneous active sites, and severe charge recombination, single-atom incorporation within MOFs offers clear catalytic advantages. Strategies such as metal-node anchoring, linker metallation, pore confinement, and defect engineering enable precise control over atomic dispersion, electronic structure, and charge-transfer pathways, while MOF-derived and composite systems further enhance conductivity and light utilization. These findings underscore the importance of rationally designing and combining anchoring strategies to maximize photocatalytic efficiency. Nevertheless, challenges related to limited solar-light utilization, framework stability under prolonged irradiation, and high-density single-atom stabilization remain. Addressing these issues will require advanced *in situ* and *operando* characterization, coupled with theoretical modeling, to establish reliable structure–activity relationships. Overall, MOF-based single-atom photocatalysts represent a promising platform for efficient and sustainable solar-driven CO_2_ conversion.

## Author contributions

Adnan Majeed: writing the original draft and software. Minh-Khoa Duong: writing, review and editing. Van-Duc Nguyen: data curation. Trong-On Do: conceptualization, resources, supervision, and overall guidance.

## Conflicts of interest

The authors declare no conflicts of interest.

## Data Availability

No primary research results, software, or code have been included, and no new data were generated or analyzed as part of this review.
